# Acute Toxicity Assays with Adult Coral Fragments: A Method for Standardization

**DOI:** 10.3390/toxics12010001

**Published:** 2023-12-19

**Authors:** David Brefeld, Valentina Di Mauro, Matthias Y. Kellermann, Samuel Nietzer, Mareen Moeller, Laura H. Lütjens, Sascha Pawlowski, Mechtild Petersen-Thiery, Peter J. Schupp

**Affiliations:** 1 Environmental Biochemistry Group, Institute for Chemistry and Biology of the Marine Environment (ICBM), School of Mathematics and Science, Carl von Ossietzky University of Oldenburg, Ammerländer Heerstraße 114-118, 26129 Oldenburg, Germany; valentina.di.mauro@uol.de (V.D.M.);; 2 Department of Product Safety, Regulatory Ecotoxicology, BASF SE, Carl-Bosch-Straße 38, 67056 Ludwigshafen am Rhein, Germany; 3 Product Stewardship and EHS Data Management, BASF Personal Care and Nutrition GmbH, Rheinpromenade 1, 40789 Monheim am Rhein, Germany; 4 Helmholtz Institute for Functional Marine Biodiversity (HIFMB), University of Oldenburg, Ammerländer Heerstraße 231, 26129 Oldenburg, Germany

**Keywords:** scleractinia, ecotoxicology, test method, personal care products, toxicity, standardization, UV filter, pesticides

## Abstract

Coral reefs are globally declining due to various anthropogenic stressors. Amongst those, chemical pollutants, such as pesticides from agricultural runoff, sewage or an overabundance of personal care products in coastal waters due to intense tourism, may be considered as a local stressor for reef-building corals. The extent to which such chemicals exhibit toxic effects towards corals at environmentally relevant concentrations is currently controversially discussed and existing studies are often based on varying and sometimes deficient test methods. To address this uncertainty, we adapted available methods into a reliable and comprehensive acute coral toxicity test method for the reef-building coral *Montipora digitata*. The toxicities of the four substances benzophenone-3 (BP-3), Diuron (DCMU), copper (Cu^2+^ as CuCl_2_, positive control) and dimethylformamide (DMF, solvent) were assessed in a 96 h semi-static test design. Endpoints such as maximum quantum yield, bleaching, tissue loss and mortality were evaluated with respect to their suitability for regulatory purposes. Overall, the endpoints bleaching and mortality yielded sensitive and robust results for the four tested substances. As the test method follows the principles of internationally standardized testing methods (ISO, OECD), it can be considered suitable for further validation and standardization. Once validated, a standardized test method will help to obtain reproducible toxicity results useful for marine hazard and risk assessment and regulatory decision making.

## 1. Introduction

Coral reefs, some of the most diverse and fragile ecosystems on Earth, are currently facing a significant decline from anthropogenic factors such as climate change, overfishing and marine pollution. These stressors pose a substantial threat to the health of coral reefs worldwide [1] and the valuable ecosystem services they provide are dramatically changing and might even vanish in the future [2,3,4]. 

Anthropogenic factors such as agricultural runoff, wastewater discharge and/or coastal recreational activities are common sources of pesticides, pharmaceuticals and personal care products containing substances that are potentially harmful to corals (e.g., ultraviolet (UV) filters, polycyclic aromatic hydrocarbons (PAHs)) [5,6]. Since corals have not yet been considered as standard organisms for aquatic toxicity testing, the evaluation and comparison of toxicity thresholds is extremely challenging, resulting in large uncertainty regarding the toxicity of many substances that potentially enter the coral reef environment [7,8].

Although a scientific basis has not yet been established, first efforts have been initiated, for example, in the islands of Hawaii (SB2571, https://www.capitol.hawaii.gov/sessions/session2018/bills/SB2571_.HTM, accessed on 8 December 2023) and Palau (SB10-135, https://www.palaugov.pw/wp-content/uploads/2018/10/RPPL-No.-10-30-re.-The-Responsible-Tourism-Education-Act-of-2018.pdf, accessed on 8 December 2023) to regulate the use of substances such as organic UV filters in sunscreens, with the aim to protect local coral reefs [9]. However, these bans are based on limited toxicity data, and the directed shift towards inorganic UV filters is not without controversy. Therefore, a standardized test system for corals is urgently needed, particularly in regard to sound regulatory decision making and the formulation of evidence-based policies [7,8,9]. To fill this critical knowledge gap, it is imperative to obtain reliable and reproducible data using a standardized test system [7,8,10,11,12]. Such a test system should build upon existing test guidelines (e.g., OECD Test No. 202 [13] and OECD Test No. 203 [14]) while incorporating endpoints able to assess the physiological responses of corals to potentially harmful chemical substances. 

Endpoints that allow a reliable toxicity assessment of potentially harmful substances should be clearly defined for the test species and ecologically relevant at the population level [15,16]. The most severe endpoint that is commonly assessed in acute toxicity testing is mortality (e.g., [10,17,18,19]). As it is relevant to any test animal, it allows the comparison of toxicity estimates among species, indicating their relative sensitivity to the substance. Since the mortality endpoint merely captures the most severe reaction to a substance, many relevant sub-lethal endpoints are commonly used in toxicity studies with corals. These endpoints include the assessment of tissue loss/necrosis, the determination of coral bleaching and the measurement of maximum quantum yield (Fv/Fm) [7,8]. Tissue loss can be defined as the sloughing or necrosis of coral tissue that reveals the calcareous skeleton underneath and is a relatively severe reaction that usually preempts mortality [20]. Corals living in symbiosis with dinoflagellates of the family *Symbiodiniaceae* commonly exhibit a stress response known as bleaching, where the expulsion of their symbiotic partners leads to discoloration of the coral tissue [21]. Bleaching over a prolonged period of time can lead to starvation of the coral host and may result in more severe reactions, such as tissue loss and mortality [22]. The photosynthetic efficiency and health of the symbiotic dinoflagellate algae can be assessed by measuring their maximum quantum yield (Fv/Fm) [23,24]. As the coral host relies on the metabolic products of its symbiotic partners for nourishment, a reduction in photosynthetic efficiency or damage to the photosystems can have harmful effects on the coral holobiont [24,25,26]. Thus, non-standardized endpoints such as photochemical and physiological examination of corals are critical parameters, representing fast and reliable indicators for the health status of the coral. By incorporating these endpoints into a test method, a comprehensive evaluation of the toxicity of a substance towards corals can be achieved, considering both non-standardized endpoints (e.g., physiological health of zooxanthellae) and the overall survival and well-being of the coral holobiont. Many of these endpoints have previously been integrated into ecotoxicological test methods for corals to assess the toxicity of various substances [6,7,8,15,27]. However, their composition and the utilized experimental setups are often highly variable, constraining the comparison and reliability of results [7,8,15]. 

Although detailed water analyses quantifying the actual exposure concentration of the test chemicals are crucial for a comprehensive toxicity assessment, they have often been omitted in previous ecotoxicological studies [8]. However, these data should accompany any aquatic ecotoxicological assay to establish comparability of ecotoxicological results.

In this study, we built upon the existing literature and integrated methods into an acute toxicity test system designed for adult fragments of the scleractinian coral *Montipora digitata*. The test method was tested using a variety of model substances (benzophenone-3 (BP-3), Diuron (DCMU) and copper(II)chloride (CuCl_2_)), as well as the carrier solvent dimethylformamide (DMF). It provides unambiguous assessments of non-standardized and standardized endpoints, yielding reliable results. We aim at contributing to the growing body of knowledge surrounding coral toxicity studies and at facilitating the standardization of toxicity tests for corals that enable evidence-based decision making in regulatory frameworks.

## 2. Materials and Methods

### 2.1. Preparation of M. digitata Fragments

**Figure 1:**
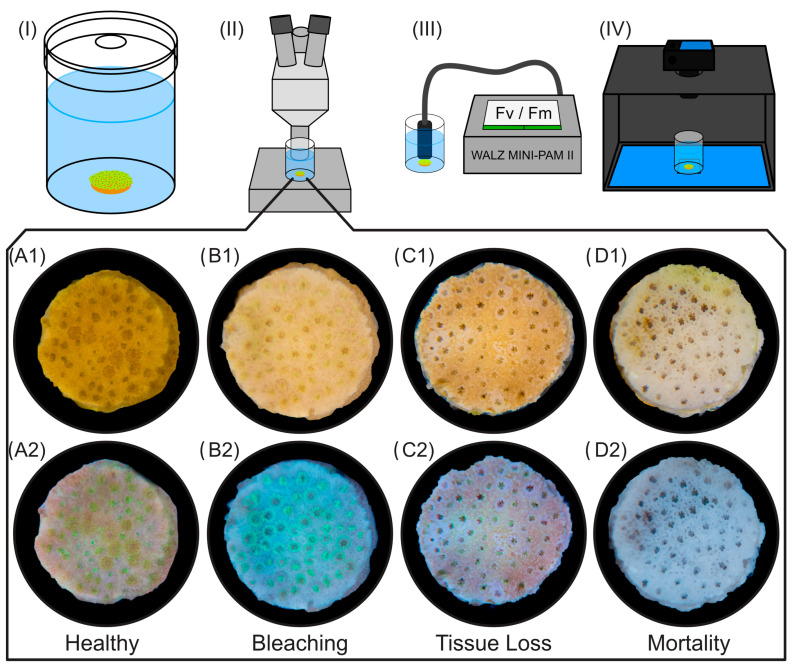
(**I**) *Montipora digitata* fragment inside test vessel and endpoint assessments using (**II**) microscopy, (**III**) maximum quantum yield (Fv/Fm) measurements and (**IV**) imaging with a photo box. The box underneath shows microscopy images of fragments exposed to Cu^2+^ under (row 1) white light and (row 2) blue light. Fragment (**A1**,**A2**) is healthy and shows retracted and extended polyps. Fragment (**B1**,**B2**) shows strong bleaching with increased fluorescence brightness. Fragment (**C1**,**C2**) shows tissue loss between debilitated polyps and partial mortality. Fragment (**D1**,**D2**) shows complete mortality.

*Montipora digitata* (Dana, 1846) is a reef-building coral species that is common in shallow reef habitats in the Indo-Pacific area [28]. The colonies usually grow digitate or arborescent [28,29] but if in contact with smooth, hard surfaces they can also develop an encrusting growth type (own observation). Mother colonies used to obtain fragments for this study were cultivated in a 6000 L recirculating system with stable water parameter conditions (Supplementary Materials S1.1). Leveraging the plate-like growth type of *M. digitata*, large numbers of uniform disc-shaped fragments with a diameter of about 10 mm were produced using a drill press equipped with a diamond core drill bit. After drilling, the discs were glued onto a glass plate using a drop of cyanoacrylate glue (Spezial 483-HV dickflüssig, 2construct GmbH & Co. KG, Ulm, Germany), followed by a recovery phase of three weeks. For that purpose, the glass plates with the glued-on fragments were placed inside a recirculating aquaculture system under artificial lighting (Radion^®^ G6 LED, EcoTech^®^ LLC, Bethlehem, PA, USA). The light source was adapted to resemble the light in the ecotoxicological test setup in terms of spectrum and intensity (about 80 μmol m^−2^ s^−1^) to acclimate the coral fragments to the testing conditions and prevent light-induced bleaching. Twelve hours of constant illumination were followed by 12 h of complete darkness to form a diurnal pattern. Between light and dark phases, half-hourlong ramps from 0 to 100% light intensity and vice versa were set to simulate sunrise and sunset. All fragments were obtained from clones of the same mother colony to reduce potential heterogeneity due to genetic variability. The upward-facing discs (Figure 1) allow convenient and reliable measurement of the experimental endpoints described below. More information about the coral-cultivating conditions can be found in Supplementary Materials S1.1. 

### 2.2. Endpoint Assessments

To assess the effect of the test substances on *M. digitata* fragments, several biological endpoints were examined. Endpoints were selected to evaluate concentration-dependent effects on both the coral animal host and its symbiotic algae. 

**Maximum quantum yield (Fv/Fm):** The maximum quantum yield (Fv/Fm) was measured at the start (t_0_) and end (t_96_) of the experiment, using a WALZ MINI-PAM-II (Heinz Walz GmbH, Effeltrich, Germany). This allowed the calculation of the loss in Fv/Fm over the course of the experiment while considering the heterogeneity among fragments. To ensure consistent measurements, the fiberoptic (ø 5.5 mm) of the PAM was equipped with a cylindrical attachment used as spacer (Supplementary Materials S1.2 —Figure S1) that made it possible to gently place the fiberoptic onto the fragments with minimal variation in distance (approx. 7 mm). Iz`n consequence, Fv/Fm could always be measured with the same settings (measuring light intensity = 7, frequency = 3; saturation pulse intensity = 10, width = 0.6). The fiberoptic covered most of the fragment’s surface and a single Fv/Fm measurement per fragment was taken as their dark adaptation was lost after the saturation pulse. The loss in Fv/Fm was calculated for each fragment individually by subtracting its Fv/Fm at the end from its Fv/Fm at the beginning of the experiment. 

**Bleaching:** Bleaching was determined from photographs of the fragments taken under identical light conditions at the beginning and at the end of the experiment. The average pixel intensities of the fragments were measured as a proxy for their chlorophyll content and symbiodinium density (cf. [30,31]), and an increase in the average pixel intensities was regarded as a loss of chlorophyll or symbiodinium cells. Hence, the change in the fragments’ average pixel intensities over the course of the experiment denoted the bleaching intensity. To achieve identical photographing conditions, an enclosed photo box with integrated natural white (5500 K) LED lighting was used. The bottom of the photo box was of light-blue color to achieve good contrast to the fragments, and a placement indicator was used to ensure that the test vessels were always placed at the same position. The camera (EOS M3 with EF-M 15–45 mm lens, Canon Inc., Tokyo, Japan) was set to record with the same settings (45 mm, F10, 1/200 sec, ISO 400, saved as raw file), making the photographs comparable to each other. A color reference card (ColorChecker Classic Nano, Calibrite LLC, Wilmington, DE, USA) was photographed at the beginning of each photographing session to allow for post hoc color calibration of all images and to achieve device independency that makes photographs captured with different cameras comparable [32]. The software darktable (v4.0.0, darktable Team, Saint Petersburg, Russia, www.darktable.org, accessed on 8 December 2023) was used to apply the color correction to the ColorChecker reference image and to copy the corrections over to the other images. The color intensities of the fragments were calculated using the image analysis software ImageJ [33] (v1.54c; Fiji distribution [34]). An ImageJ macro program was developed to separate the fragments from the background and measure their average greyscale intensity on a scale from 0 to 255. The ImageJ macro program and a comprehensive workflow of the image post-processing and subsequent analyses can be found in Supplementary Materials S2. The bleaching intensity was calculated for each fragment individually by subtracting its average pixel intensity at the beginning of the experiment from its average pixel intensity at the end.

**Tissue loss and mortality:** Tissue loss was defined as the disintegration of tissue between the polyps and the exposure of the coral skeleton. Concurrently, the polyps will retract deep into the skeleton and appear darker in color. Eventually, the coral polyps will disintegrate, leading to their death (mortality). Tissue loss and mortality were evaluated at the end of the experiment using a stereomicroscope (GSZ type, Carl Zeiss, Jena, Germany) and assessed on a binary (yes/no) scale. To assess the health status of the corals, a light source was used to illuminate the fragments (Neopixel Model SK6812RGBW-BW, Adafruit Industries LLC, New York, NY, USA) that was switchable between cool white (6000–7000 K) and blue (460–470 nm) light. The blue light excites the fluorescence of *M. digitata*, which is useful for determining the health of the individual. The white light is most useful to detect tissue loss due to the exposure of the white-appearing carbonate skeleton. A single-use pipette was applied to blow a gentle stream of water across the polyps that blew off any dead, necrotized tissue. Colony mortality was judged as definitive if at least 50% of polyps disintegrated. Figure 1 shows representative images of the assessed primary endpoints (i.e., bleaching and tissue loss/mortality).

**Morphological/behavioral abnormalities:** In addition to the previously mentioned endpoints, any additional findings such as ejection of their mesenterial filaments and/or deep retraction of the polyps into the skeleton as well as widely open polyps’ mouths, that may occur during the exposure of the coral (polyp debilitation), were observed and noted as secondary endpoints (Supplementary Materials S1.3—Figure S2).

### 2.3. Preparation of Stock Solutions and Test Concentrations

Screw-top bottles (0.5–2 L, borosilicate glass) and test vessels (100 mL beakers without spout, ø 45 mm, borosilicate glass) were cleaned and heat-treated in a muffle furnace at 550 °C for at least 5 h to eliminate potential organic contaminants. Pharmaceutically pure artificial sea salt (Pro-Reef Sea Salt, Tropic Marin^®^ AG, Hünenberg, Switzerland) and de-ionized (reverse osmosis) water were used to produce artificial seawater (ASW) with a salinity of approx. 34 PSU. Seawater was prepared at least 24 h in advance to ensure that the salts had fully dissolved. Alkalinity (KH) was measured using titration tests (KH/Alkalinity Profi Test, Salifert, Duiven, the Netherlands) and adjusted to about 8–8.5 °dKH using NaHCO_3_ to ensure adequate pH buffering capacity. Stock solutions were prepared at least 48 h before the start of the assays by stirring inside an incubator at 26.5 ± 1.5 °C using magnetic stir bars. To remove undissolved particles, stock solutions were left to settle for at least one hour before carefully transferring about 80% from the center portion of the volume to a new bottle using volumetric glass pipettes. Test concentrations were prepared on the day of the experiment by diluting highly concentrated stock solutions of the respective test substance with ASW.

**Copper (II) (Cu^2+^).** An 8 mg L^−1^ CuCl_2_ (3.78 mg L^−1^ Cu^2+^) stock solution was made by pipetting 0.08 mL of a highly concentrated CuCl_2_ storage solution (100 g L^−1^ CuCl_2_ in ultrapure water; made up from CuCl_2_·2H_2_O; ThermoFisher GmbH (Kandel, Germany), 99% purity, CAS 10125-13-0) into each liter of ASW (without significantly changing its salinity). Seven test concentrations were prepared with dilution factor 2 at nominal 0.01, 0.02, 0.04, 0.08, 0.16, 0.32, 0.64 mg L^−1^ CuCl_2_. The theoretical Cu^2+^ content of dissolved CuCl_2_ in water is approximately 47.3%. Hence, the test concentrations were expected at nominal concentrations of 0.005, 0.009, 0.019, 0.038, 0.076, 0.151 and 0.303 mg L^−1^ Cu^2+^.

**Benzophenone-3 (BP-3).** A saturated BP-3 stock solution was prepared by adding 12 mg of 2-hydroxy-4-methoxybenzophenone (BP-3; Sigma-Aldrich Chemie GmbH (Steinheim, Germany), 98% purity, CAS 131-57-7) to each liter of seawater. The saturation point was expected at approx. 9 mg L^−1^ BP-3 as derived from pre-experimental data. Six test concentrations were prepared in a linear range at nominal 7, 8, 9, 10, 11 and 12 mg L^−1^. A linear concentration range was chosen based on the results of pilot experiments. The highest tested BP-3 concentration was the saturated stock solution.

**3-(3,4-Dichlorphenyl)-1,1-dimethylurea (DCMU/Diuron).** A saturated DCMU stock solution was prepared by adding 50 mg of DCMU (Diuron; Sigma-Aldrich Chemie GmbH (Steinheim, Germany), ≥98% purity, CAS 330-54-1) to each liter of ASW. The saturation point was expected at approx. 30 mg L^−1^ DCMU as derived from pre-experimental data. Nine test concentrations were prepared with dilution factor 5 at nominal 0.0001, 0.0006, 0.003, 0.016, 0.08, 0.4, 2, 10 and 50 mg L^−1^. The highest DCMU concentration was the saturated stock solution. The relatively high dilution factor was used because non-lethal and lethal endpoints were expected to occur at drastically different concentration ranges (based on pre-experiments).

**N,N-dimethylformamide (DMF).** A 4720 mg L^−1^ stock solution was made by adding 5 mL of DMF (HiPerSolv CHROMANORM; Avantor Performance Materials Poland S.A. (Gliwice, Poland), ≥99.9% purity, CAS 68-12-2, density (DMF) = 944,000 mg L^−1^) to 995 mL of ASW. Six test concentrations were prepared with dilution factor 3 at nominal 5.83, 17.48, 52.44, 157.33, 472 and 1416 mg L^−1^. DMF was tested to estimate its effects on the test organism when used as a carrier solvent, so no chemical control analyses were performed.

For all experiments, negative controls of substance-free ASW were prepared. For the BP-3, DCMU and DMF experiments, a positive control with a concentration of 0.08 mg L^−1^ CuCl_2_ (0.038 mg L^−1^ Cu^2+^) was used (prepared as described above). To prevent coral bleaching due to nutrient limitation, the test solutions and controls were enriched with 0.025 mg L^−1^ NH_3_/NH_4_^+^ (using NH_4_Cl) and 0.025 mg L^−1^ PO_4_^3−^ (using KH_2_PO_4_). The pH in each concentration and control was measured using a pH probe (WTW Multi 3630 IDS with SensoLyt 900, Xylem Analytics Germany Sales GmbH & Co. KG, Weilheim, Germany) and adjusted to pH 8–8.3 using either NaOH (1 M) or HCL (1 M). Per test concentration and control (ASW, Cu^2+^), six (*n* = 6) test vessels were filled with 80 mL of the corresponding solution using dispensers (Beatus, Fisherbrand™, Fisher Scientific GmbH, Schwerte, Germany). After filling, the test vessels were covered with a glass lid (ø 50 mm) that had a hole (ø 10 mm) for balancing gas exchange and evaporation (Figure 1) and placed inside an incubator at 26.5 °C.

### 2.4. Experimental Setup and Procedure

At the beginning of the assay (0 h, t_0_), *M. digitata* fragments were removed from the glass plates without injury and transferred with undue delay into an incubator (Lovibond TC445 S/455 L/2–40 °C, Lovibond-Tintometer GmbH, Dortmund, Germany) set to an air temperature of 26.5 ± 1.5 °C. Fv/Fm baseline measurements of each fragment were taken after a dark adaptation period of at least one hour. 

Afterwards, the fragments were placed individually inside their corresponding vessels, and baseline photos of each fragment were taken. All beakers were then randomly placed on trays and placed inside the incubator. LED lighting (Radion^®^ G6, EcoTech^®^ LLC, Bethlehem, PA, USA) set to an intensity of approx. 80 μmol m^2^ s^−1^ was used for illumination in a 12 h:12 h light:dark cycle. The applied light spectrum and the device settings can be found in Supplementary Materials S1.4 (Figure S3). Using a lubricant-free air compressor (MEDO LA-45B, Nitto Kohki Europe GmbH, Steinenbronn, Germany), the air inside the incubator was constantly replenished to prevent any changes in CO_2_/O_2_ within the incubator as well as to remove potential aerosols. The general water parameter conditions during exposures closely followed the culture conditions of the mother colonies (Supplementary Materials S1.1). Temperature/light loggers (HOBO^®^ Pendant temp/light, Onset Computer Corporation, Bourne, MA, USA) were used to log the exact air temperature within the incubator and to make sure the light setup was applied correctly. The mean temperature condition was held within 26.5 ± 1.5 °C (Supplementary Materials S1.5—Tables S1 and S2). Additional water parameters O_2_, pH, salinity and KH remained stable during exposure (Supplementary Materials S1.5—Tables S1 and S2) and were measured using a WTW Multi 3630 IDS with probes for oxygen concentration (FDO^®^ 925), pH (SensoLyt 900) and salinity (TetraCon 925) (Xylem Analytics Germany Sales GmbH & Co. KG, Weilheim, Germany). All probes were calibrated shortly before the experiment. KH was measured using titration tests (Profi KH/Alk test, Salifert, Duiven, The Netherlands).

After approx. 48 h (t_48_), the control and test concentrations were replaced to ensure adequate substance retention and to maintain good water quality as performed in a semi-static test system. To achieve a seamless exchange of solutions, a new set of testing vessels was filled with “fresh” test concentrations (prepared as described in Section 2.3) and the fragments were carefully transferred to the new vessels. The vessels were randomized again and placed back into the incubator. Water samples for chemical control analysis were taken as described in Section 2.4.1, and water quality parameters (salinity, O_2_, pH, KH) were measured in all aged and fresh controls and test concentrations as described above.

After 96 h (t_96_), the experiment was finalized by measuring the Fv/Fm (at the end of the dark phase), taking photos of the fragments and assessing tissue loss, mortality and behavioral abnormalities under the microscope. Water samples were taken as described in Section 2.4.1, and water parameters were measured in each test vessel as described above. 

#### 2.4.1. Water Sampling

Water sampling for UPLC-MS and ICP-OES analyses was always conducted following the same scheme. For UPLC-MS analyses, 5 mL of sample was transferred into 20 mL glass scintillation vials (986542, Wheaton, DWK Life Sciences GmbH, Wertheim am Main, Germany). For ICP-OES analyses, 20 mL of sample was passed through 0.2 µm Nylon syringe filters (5191-5910, Agilent, Santa Clara, CA, USA) into 30 mL screw-cap tubes (62.543, Sarstedt AG & Co. KG, Nümbrecht, Germany) in duplicates. All samples were frozen immediately and stored at −20 °C until analysis. At the beginning of the experiment (t_0_), UPLC-MS samples were taken from each test concentration and the negative control in triplicates. ICP-OES samples were taken from the negative control, positive control and highest test concentration. During the water exchange (t_48_), UPLC-MS samples were taken from the “fresh” test concentrations and negative control in triplicates. After the coral fragments were removed from the test vessels, further UPLC-MS samples were taken from all replicates of each test concentration and the negative control. Then, ICP-OES samples were taken from all replicates of the positive control and from every second replicate of the negative control and the highest test concentration. At the end of the experiment (t_96_), UPLC-MS and ICP-OES samples were taken from the test vessels as described above (t_48_).

#### 2.4.2. Validation Criteria

To ensure good water quality and adequate retention of the test substance, specific validation criteria were set (Table 1). Slight bleaching and a loss of photosynthetic efficiency regularly occur in the negative control group of *M. digitata*. A bleaching rate of less than 30% and an initial Fv/Fm value of ≥0.450 were considered adequate. There should be no mortality, and tissue loss should not occur on more than one fragment in the negative control (≤20%). The test should always be accompanied by suitable chemical control analyses to determine the actual test substance concentrations. The recovery of the mean measured test concentrations should be within the range of 80–120% of the nominal concentration. If the recovery is within this range, the values can be displayed as nominal, otherwise they are displayed as mean measured concentrations. 

### 2.5. Chemical Control Analyses

Chemical control analyses of the water samples (cf. Section 2.4.1) were performed to establish actual concentrations of test concentrations over the course of the experiment. 

#### 2.5.1. Analyses of Organic Test Substances: BP-3 and DCMU

Recoveries of the organic test substance BP-3 and DCMU were analyzed using vortex-supported liquid–liquid microextraction followed by UPLC-MS analysis.

**Extraction method**: The vortex-supported microextraction method was adapted from Miller et al. [11] and modified for the extraction of organic compounds dissolved in seawater media. In brief, the seawater samples (cf. Section 2.4.1) were acidified with formic acid (FA; Biosolve Chimie SARL, Dieuze, France), ULC-MS grade, 99% purity, CAS 64-18-6) and extracted with the highly lipophilic organic extractant tetrachloroethylene (PCE; Honeywell Specialty Chemicals Seelze GmbH (Seelze, Germany), HPLC grade, ≥99.9% purity, CAS 127-18-4). For DCMU, a disperser solvent, namely, ethyl acetate (EtOAc; VWR International bvba (Leuven, Belgium), HPLC grade, ≥99.8% purity, CAS 141-78-6), in a 1:1 ratio with PCE had to be added to increase its extraction efficiency. For validation of the extraction efficiency, an internal standard (IS; deuteride BP-3 as BP3-*d*_5_ and deuteride DCMU as DCMU-*d*_6_ (Sigma-Aldrich Co. (St. Lois, MO, USA), analytical standard, CAS 1219798-54-5, and CAS 1007536-67-5, respectively) was applied to the water samples before PCE extraction. For measuring the BP-3 and DCMU concentrations, an aliquot of the formed PCE droplet was transferred, 100% reduced and refilled with MeOH (MeOH; Biosolve Chimie SARL (Dieuze, France), ULC-MS grade, >99.98% purity, CAS 67-56-1) in 2 mL amber glass vials (neochrome ND9, neoLab Migge GmbH, Heidelberg, Germany). The resulting concentrations were further processed to fit into the analytical range of the instrument. To quantify the BP-3 and DCMU test concentrations, an external (solvent) linear calibration curve made from an inhouse BP-3 and DCMU standard (100% diluted in MeOH) was used, with a five-step dilution series (i.e., 400, 200, 100, 50, 25 (µg L^−1^)) for BP-3 and an 11-step dilution series (i.e., 500, 100, 50, 10, 5, 1, 0.5, 0.1, 0.05, 0.01, 0.005 (µg L^−1^)) for DCMU. Performing a calibration and method validation, quality controls (QCs; *n* ≥ 3) for all target compounds (i.e., BP-3, BP3-*d*_5_, DCMU and DCMU-*d*_6_) were included and highlighted %RSD < 3 with R^2^ > 0.99 for the calibration curve and %RSD < 6 for the extraction method with a valid mean recovery of 105% (BP3-*d*_5_) and 57% (DCMU-*d*_6_) (Supplementary Materials S1.6 (including Tables S3−S10; Figures S4 and S5)). Carryover of 0.0005–0.0008 mg L^−1^ for BP-3 during analysis did not affect the analytical results while being under the limit of detection (LOD = 0.02 mg L^−1^) and the limit of quantification (LOQ = 0.07 mg L^−1^) of the calibration curve (Tables S7−S10). For DCMU, the calibration curve resulted in LOD = 0.0006 mg L^−1^ and LOQ = 0.0017 mg L^−1^ (Table S7). 

UPLC-MS analyses: Chromatographic analyses of the extracted samples were performed by WATERS ACQUITY H Class Plus ultraperformance liquid chromatography (UPLC) connected to a micro tandem quadrupole (TQ) mass spectrometer (MS) (Waters Xevo TQ-S micro, Waters Co., Milford, MA, USA). The total run time of the UPLC-MS system was 7 min for BP-3 and 8 min for DCMU, with a constant flow rate of 0.2 mL min^−1^ that passed through an ACQUITY UPLC BEH C18 column (1.7 µm; 2.1 × 150 mm). The separated compounds were directed into an MS system through an electron spray ionization source set to positive ion mode (ES+). The method condition of the MS system was adjusted using Waters instruments integrated software MassLynx IntelliStart (v4.2, Waters Co., Milford, MA, USA) performing multiple reaction monitoring (MRM). Further details of the extraction method and the analytical performance are described in Supplementary Materials S1.6.

#### 2.5.2. Analyses of Inorganic Test Substance: CuCl_2_

Cu^2+^ concentrations in the seawater samples were analyzed using inductively coupled plasma optical emission spectroscopy (ICP-OES, at Dr. Biener GmbH, Wartenberg, Germany). The seawater samples were sent away for analysis shortly after acquisition and without further processing. 

During the same analysis, a comprehensive analysis of the measurable inorganic constituents of the seawater was conducted for the negative control and the highest test substance concentrations to elucidate potential interaction among the test substance and the elements in the seawater. 

### 2.6. Data Analysis

Data entry was accomplished using Excel (v2021, Microsoft Corporation, Redmond, WA, USA) and data analyses were performed using R (v4.2.0, R Foundation for Statistical Computing, Vienna, Austria) with the RStudio IDE (v2022.12.0, Posit Software PBC, Boston, MA, USA). Data manipulation, summarizing and plotting were performed using tidyverse [35] (v2.0.0). Cumming estimation plots were produced using the dabestr package [36] (v0.3.0). 

The obtained Fv/Fm loss and bleaching intensity values were min-max transformed to a scale from 0 to 100 using the formula

(1) $$X_{scaled}=\left(\frac{x-x_{min}}{x_{max}-x_{min}}\right)*100$$

where *x* is either the measured Fv/Fm loss or the bleaching intensity value. 

Differences in the mean Fv/Fm loss and the mean bleaching intensity among test concentrations were evaluated using one-way analysis of variance (ANOVA). Model assumptions for homogeneity of variance and normality were checked visually using residual diagnostics plots and statistically using Shapiro–Wilk normality tests and Levene’s or Fligner–Killeen tests for homogeneity of variance (the latter was used if residuals were non-normal). If the ANOVA was statistically significant (*p* < 0.05) and model assumptions were satisfied, Dunnett’s tests were carried out post hoc using the multcomp package [37]. The car [38] (v3.1-2) and sandwich [39,40] (v3.0-2) packages were used to utilize heteroscedasticity consistent covariance estimation (HC3) for the computation of ANOVA and Dunnett’s tests where appropriate. If model assumptions were not satisfied, non-parametric one-way permutation ANOVAs (PANOVAs) were computed instead, using the coin package [41] (v1.4-2). If statistically significant (*p* < 0.05), pairwise post hoc tests were performed that compared the mean Fv/Fm or bleaching intensity of each test concentration to the negative control. *p*-values were adjusted for multiple comparisons using the Holm method [42]. Differences in the probability of tissue loss and mortality between the control and all treatment concentrations were evaluated using pairwise Fisher’s exact tests on 2 × 2 contingency tables. If any pairwise tests were significant (*p* < 0.05), EC_50_ (for Fv/Fm loss, bleaching, tissue loss) and LC_50_ (for mortality) values were calculated using the drc package [43] (v3.0-1). Dose–response models (DRMs) were fitted using different underlying distributions (natural-logistic, logistic, log-logistic, generalized log-logistic, Weibull) and varying parameterizations. Four or five parameters without lower/upper limits were considered for continuous data (Fv/Fm loss, bleaching). Two parameters, where the lower limit was fixed at 0 and the upper limit was fixed at 1, were considered for binomial data (tissue loss, bleaching, morphological abnormalities). The models with the best fit (lowest Akaike information criterion) were chosen for the calculation of the EC_50_/LC_50_ values. The satisfaction of model assumptions was checked using residual diagnostics plots and statistical tests. 

For the chemical analytical verification, LOD and LOQ were calculated using the equations

(2) $$LOD=\left(\frac{3.3\cdot \delta_{SD\;residuals}}{s}\right)$$

and

(3) $$LOQ=\left(\frac{10\cdot \delta_{SD\;residuals}}{s}\right)$$

where *δ* is the SD of the residuals, and s is the intercept of the regression curve. 

Recoveries were calculated using the following equation:

(4) $$\%Rec=\left(\frac{{concentration}_{actual}}{{concentration}_{target}}\right)\cdot 100$$

## 3. Results

The general water chemistry parameters (i.e., salinity, O_2_, pH, calcium and alkalinity) were within the acceptable limits of the validation criteria for water parameters of the negative control group throughout the experiments, as shown in Supplementary Materials S1.5 (Tables S1 and S2).

### 3.1. Recovery of the Test Substances

When comparing the mean measured concentrations of the test substances to their nominal concentrations, the average recoveries were located outside the validation range of 80–120%. They ranged from 44.1–75.6% for BP-3, 61.7–139.1% for DCMU and 20.9–40% for Cu^2+^ (Table 2). To acquire valid estimates of the substances’ toxicities, results are based on mean measured concentrations instead of nominal concentrations where applicable.

In the first phase (t_0_–t_48-aged_) of the semi-static renewal system, the recoveries of the test concentrations were ranging from 73.1–79.5% for BP-3, 86.5–111.5% for DCMU and 100–177.8% for Cu^2+^ (Table 2). In the second phase (t_48-fresh_–t_96_), 77.3–90.5% of BP-3, 88.1–106.6% of DCMU and 77.8–114.3% of Cu^2+^ could be recovered (Table 2). Comparing the first and second renewal phases, the reduction rates of the test substances from the exposure waters were nearly constant except for a few outliers. Hence, on average, the recoveries of test substances within both semi-static renewal phases were ranging from 75.2–82.0% for BP-3, 89–108.4% for DCMU and 95.2–127.8% for Cu^2+^ (Table 2). DCMU was the most stable substance with average recoveries inside the pre-determined recovery range of 80–120%.

ICP-OES analysis for Cu^2+^ was challenging and resulted in lower-than-expected recoveries. It is likely that the administered CuCl_2_ reacts with the constituents of the seawater to form bonds and complexes other than Cu^2+^ [44], which might not be detectable with the analytical method. Since it can be expected that the administered chemical is present in one form or another, the results of the experiment are presented as nominal concentrations. However, the estimated effect concentrations for Cu^2+^ are limited by the uncertainty in the water analytics and need to be handled with due care. 

### 3.2. Cu^2+^ Toxicity

**Figure 2:**
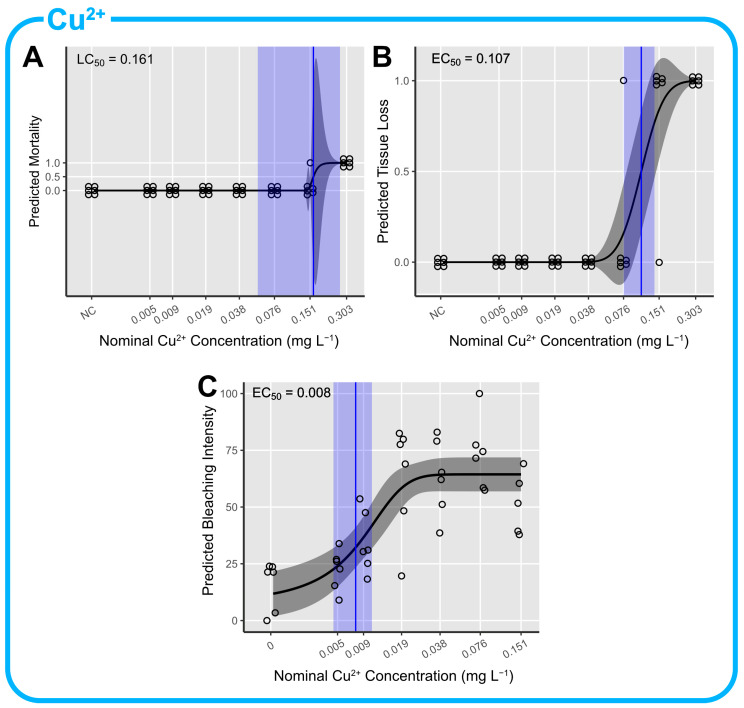
Concentration–response predictions of *M. digitata* fragments exposed to copper (II) (Cu^2+^) for (**A**) mortality; (**B**) tissue loss and (**C**) bleaching intensity. EC/LC_50_ estimates with accompanying 95% CIs are displayed in blue.

After 96 h, one fragment (17%) was considered dead at 0.151 mg L^−1^ and all fragments (100%) were found dead at 0.303 mg L^−1^ Cu^2+^. No mortality occurred in the negative control. Tissue loss was not observed in the negative control and at Cu^2+^ concentrations ranging from 0.005 to 0.038 mg L^−1^. However, a concentration-dependent tissue loss was observed in one fragment at 0.076 mg L^−1^, in five fragments at 0.151 mg L^−1^ and in all six fragments (100%) at 0.303 mg L^−1^. Cu^2+^ induced a concentration-dependent bleaching response (ANOVA, F(6) = 13.805, *p* < 0.001) with an average bleaching intensity that ranged from 16% in the negative control to a maximum of 73% in the third highest test concentration (0.076 mg L^−1^). Cu^2+^ did not induce significant Fv/Fm loss (ANVOA, F(6) = 1.548, *p* = 0.193; Supplementary Materials S1.7—Figure S6) in the *M. digitata* fragments. There was one fragment (17%) showing polyp debilitation at a Cu^2+^ concentration of 0.076 mg L^−1^ and none of the fragments ejected their mesenterial filaments (Supplementary Materials S1.7—Figure S6). The LC_50_ was estimated at 0.161 mg L^−1^ with a 95% confidence interval from 0.548 to 0.267 mg L^−1^ (Weibull(1) two-parameter DRM, t(46) = 4.795, *p* < 0.001; Figure 2A). The EC_50_ for tissue loss was estimated at 0.107 mg L^−1^ with a 95% confidence interval from 0.076 to 0.138 mg L^−1^ (log-normal two-parameter DRM, t(46) = 4.973, *p* < 0.001); Figure 2B). The EC_50_ for bleaching was estimated at 0.008 mg L^−1^ with a 95% confidence interval from 0.005 to 0.011 mg L^−1^ (logistic three-parameter DRM, t(38) = 4.973, *p* < 0.001; Figure 2C).

Additional results can be found in Supplementary Materials S1.7 (Figure S6). 

### 3.3. BP-3 Toxicity

After 96 h, no mortality occurred in the negative control, positive control and in the two lowest test concentrations (3.087 and 4.290 mg L^−1^). Mortality was visible in two fragments (33%) at 5.434 mg L^−1^ and became more pronounced at higher test concentrations with five fragments (83%) showing mortality at 6.386 mg L^−1^ and all fragments (100%) showing mortality at higher concentrations. Tissue loss did not occur in the negative control, positive control and in the lowest test concentration of BP-3 (3.087 mg L^−1^). It was visible in only one fragment (17%) at 4.29 mg L^−1^ and became more pronounced at higher test concentrations with four fragments (67%) showing tissue loss at 5.434 mg L^−1^ and all fragments (100%) showing tissue loss at higher concentrations. BP-3 did not induce significant bleaching before fragments started dying (PANOVA, maxT = 4.6291, *p* < 0.001; no significant post hoc tests). The positive control bleached significantly (maxT = −3.155, *p* = 0.006) with an intensity of 75%. Similarly, BP-3 did not induce significant Fv/Fm loss before fragments started dying (PANOVA, maxT = 2.692, *p* = 0.034; no significant post hoc tests). One fragment (17%) ejected its mesenterial filaments in the negative control and polyp debilitation was visible in ≥75% of all fragments without full mortality at any test concentration except the negative control (Supplementary Materials S1.8—Figure S7). 

**Figure 3:**
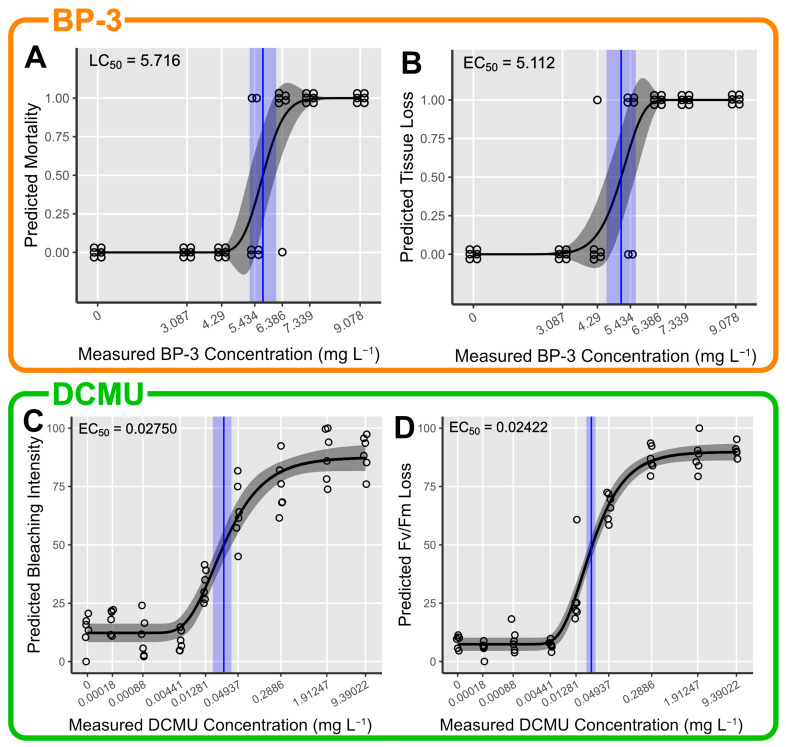
(**Top**) Concentration–response predictions of *M. digitata* fragments exposed to benzophenone-3 (BP-3) for (**A**) mortality and (**B**) tissue loss. (**Bottom**) Concentration–response predictions of *M. digitata* fragments exposed to 3-(3,4-dichlorphenyl)-1,1-dimethylurea (DCMU) for (**C**) bleaching intensity and (**D**) Fv/Fm loss. EC/LC_50_ estimates with accompanying 95% CIs are displayed in blue.

The LC_50_ was estimated at 5.716 mg L^−1^ with a 95% confidence interval from 5.256 to 6.176 mg L^−1^ (log-normal two-parameter DRM, t(40) = 24.335, *p* < 0.001; Figure 3A). The EC_50_ for tissue loss was estimated at 5.112 mg L^−1^ with a 95% confidence interval from 4.604 to 5.619 mg L^−1^ (Weibull(2) two-parameter DRM, t(40) = 21.125, *p* < 0.001; Figure 3B).

Additional results can be found in Supplementary Materials S1.8 (Figure S7).

### 3.4. DCMU Toxicity

After 96 h, mortality and tissue loss occurred in all fragments (100%) exposed to the highest test concentration (40.93958 mg L^−1^). There was no mortality and tissue loss in the negative and positive control. DCMU induced a concentration-dependent bleaching response (maxT = 3.4, *p* = 0.006) with an average bleaching intensity that ranged from 13% in the negative control to 89% in the second highest concentration (9.23022 mg L^−1^). The positive control bleached significantly with an intensity of 60% (maxT = −3.091, *p* = 0.011). Additionally, DCMU induced a concentration-dependent Fv/Fm loss in the *M. digitata* fragments (maxT = 3.5, *p* = 0.004) leading to a 90% reduction in the second highest test concentration (9.39022 mg L^−1^). The Fv/Fm loss in the positive control (13%) was not significantly higher than in the negative control (9%). One fragment (17%) showed filament ejection in the negative control and at test concentrations of 0.00088 and 0.00441 mg L^−1^. Polyp debilitation was visible in 33% of fragments at 0.01281 mg L^−1^ and in >80% at test concentrations from 0.04937 to 9.39022 mg L^−1^. (Supplementary Materials S1.9—Figure S8). No reliable LC/EC_50_ estimate could be derived for mortality and tissue loss, but the data suggest that the value lies between 9.39022 and 40.93958 mg L^−1^. The EC_50_ for bleaching was estimated at 0.02750 mg L^−1^ with a 95% confidence interval from 0.01750 to 0.03751 mg L^−1^ (Weibull(1) four-parameter DRM, t(_50_) = 6.77, *p* < 0.001; Figure 3C). The EC_50_ for Fv/Fm loss was estimated at 0.02422 mg L^−1^ with a 95% confidence interval from 0.01972 to 0.02872 mg L^−1^ (Weibull(1) four-parameter DRM, t(50) = 11.9, *p* < 0.001; Figure 3D). Additional results can be found in Supplementary Materials S1.9 (Figure S8).

### 3.5. Effects of DMF as Carrier Solvent 

**Figure 4:**
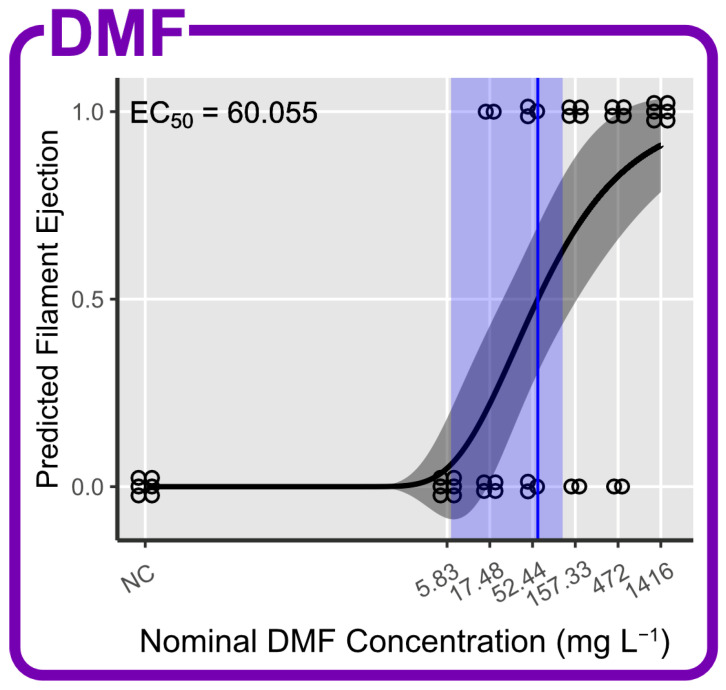
Concentration–response prediction of *M. digitata* fragments exposed to N,N-dimethylformamide (DMF) for filament ejection. EC_50_ estimates with accompanying 95% CIs are displayed in blue.

After 96 h of exposure to DMF, there was no mortality and tissue loss visible in the *M. digitata* fragments. DMF did not induce a significant bleaching response (PANOVA, maxT = 6.196, *p* < 0.001; no significant post hoc tests) and no significant Fv/Fm loss (PANOVA, maxT = 2.083, *p* = 0.253) in the range from 5.83 to 1416 mg L^−1^. The positive control bleached significantly (PANOVA, maxT = −3.093, *p* = 0.014) with an intensity of 85%. Polyps were debilitated in one fragment (17%) at 472 and 1416 mg L^−1^. Supporting information can be found in Supplementary Materials S1.10 (Figure S9) The coral fragments started to eject their mesenterial filaments at DMF concentrations as low as 17.48 mg L^−1^ (33% of fragments). The effect became more pronounced at higher concentrations with 50% of fragments showing filament ejection at 52.44 mg L^−1^, 67% at 157.33 and 472 mg L^−1^ and 100% at 1416 mg L^−1^. There were no signs of filament ejection in the negative and positive controls. The EC_50_ for filament ejection was estimated at 60.055 mg L^−1^ with a 95% confidence interval from 6.460 to 113.650 mg L^−1^ (Weibull(1) two-parameter DRM, t(40) = 2.267, *p* = 0.023; Figure 4). 

A visual example of the ejection of mesenterial filaments of DMF-treated *M. digitata* fragments can be found in Supplementary Materials S1.3 (Figure S2). An overview of LC_50_ and EC_50_ estimates for all substances and endpoints can be found in Table 3 and Supplementary Materials S1.11 (Tables S11 and S12).

## 4. Discussion

The test method established here consistently met the pre-determined validation criteria for water quality and endpoint limits in the negative control. The chosen endpoints were adequate to assess the ecotoxicological effects of substances with varying chemical and physical properties. Their objective measurement was unambiguous and repeatable. EC_50_ or LC_50_ values could be calculated for endpoints that showed significant concentration-dependent effects on the test substance (Table 3). The test organism, *M. digitata*, has proven to be adequately sensitive in the test method and is ecologically relevant as it is a common reef-building coral in the Indo-Pacific area [28]. Due to its encrusting plate-like growth, homogenous fragments can be produced that allow convenient and repeatable assessment of endpoints. In addition, mother colonies heal after fragmentation and are readily cultivated in ex situ aquaculture systems, minimizing dependence on wild-caught specimens.

Besides tissue loss and mortality, not all endpoints showed an effect for the test substances BP-3, DCMU, Cu^2+^ and DMF, suggesting that the stress response of *M. digitata* to different types of chemicals varies widely. BP-3 did not invoke a concentration-dependent Fv/Fm loss nor bleaching before fragments began to die. Under DCMU exposure, a significant Fv/Fm loss was observed along with a strong bleaching response with comparable EC_50_ estimates, while the LC_50_ was more than 300 times higher. Exposure to Cu^2+^ did not result in Fv/Fm loss but instead triggered a strong bleaching response, resulting in an EC_50_ approx. 20 times lower than the estimated LC_50_. For DMF-treated *M. digitata* fragments, none of the presented endpoints were observed in the tested concentration range, except abnormal morphological responses.

Since the EC_50_ estimates for sub-lethal endpoints (i.e., Fv/Fm loss, bleaching) might be orders of magnitude lower than the estimated LC_50_ concentrations, we suggest that these sub-lethal endpoints should be assessed along with mortality when corals are used as test organisms. As coral bleaching is considered a general stress response that is sensitive to a range of stressors [21] and has shown a similar sensitivity to Fv/Fm loss under exposure to DCMU in this study, we suggest that an acute (96 h) toxicity test for *M. digitata* includes an assessment of a bleaching and mortality endpoint. Depending on whether bleaching or mortality occurs first, the lower effect concentration should be considered for regulatory purposes. This approach prevents the toxicity of the tested substance from being massively underestimated. To verify each test, we highly recommend including at least one positive control concentration (here Cu^2+^ at 0.038 mg L^−1^, nominal or alternatively DCMU at 2.000 mg L^−1^, nominal) that consistently induces a strong bleaching response in the coral fragments. As proposed by the OECD test guidelines no. 201 [45] and 202 [13], the reproducibility/reliability of the method can additionally be verified by testing a concentration range of a reference substance that covers all endpoints (i.e., bleaching and mortality) at regular intervals. 

In standardized toxicity testing, chemical control analyses are indispensable to determine the actual test substance concentrations in both the control(s) and the treatment groups. This is of special importance if the test substance is considered as non-stable or poorly soluble in water as exposure concentrations might decline over the course of the test [46]. Although all materials that come into extended contact with the test substances were chosen to be made from inert materials (glass or fluorinated plastics), the average recovery of BP-3 over the course of the experiment fell just outside the desired range of 100 ± 20% for most concentrations with average recoveries ranging from 75% to 82%. For DCMU, the mean recoveries ranged from 89% to 108.4% and fell within the desired range, indicating that the substance remained relatively stable during exposure. Like BP-3, the recovery of Cu^2+^ (95.2–127.8%) was slightly outside the desired range. To improve the stability of the test substance during exposure, smaller water renewal intervals or even a flow-through system could be used. However, the initial nominal test concentrations for all substances could not be found at the start of each water renewal phase with values located widely outside the range of 100 ± 20%. This can be explained by the chemical property of the specific test substance. BP-3 and DCMU show relatively high LogP_OW_ values (3.6 [47] and 2.84–2.89 [48], respectively), indicating only moderate solubilities in water. Hence, higher concentrated stock solutions were prepared in this study to locate the substance- and matrix-specific solubility limits in seawater media, as no clear data existed and different seawater compositions had been used in prior test setups. The gap between nominal and resulting stock concentration in CuCl_2_ treatments additionally indicated that some active test compounds such as Cu^2+^ cations interact with the seawater matrix by forming complexes and thus are reduced from the exposure waters, although the substance was described as water soluble [49]. However, the recovery data in this study show that the substance recoveries within each water renewal phase were almost constant, leading to similar reduction rates of the test substance from the exposure waters and, thus, represent homogeneity within the test system. Chemical analytical verifications are therefore essential, especially when the effects of the exposure matrix on the test substances vary and are unknown.

Based on our water parameter analyses, we did not observe any dramatic changes in the seawater matrix (i.e., O_2_, pH, salinity, alkalinity; c.f. Supplementary Materials S1.5) after the addition of test substances that would make the medium unsuitable for an acute toxicity test by, for example, increasing the pH level. However, we highly recommend testing the seawater composition to control the test-specific seawater matrix of the control group and minimizing its intra-variances during water renewal intervals. When testing the carrier solvent DMF, *M. digitata* showed no tissue loss or mortality after 96 h in the range from 5.83 to 1416 mg L^−1^ (nominal). However, the fragments started to eject their mesenterial filaments (cf. Supplementary Materials S1.3—Figure S2) at DMF concentrations as low as 17.48 mg L^−1^ (nominal). In another study, the use of carrier solvents (e.g., DMF) in prolonged toxicity tests (16 days) led to sub-lethal effects (i.e., morphological variations) on *M. digitata* at concentrations of 100 mg L^−1^ (100 µL L^−1^) [50]. These authors collected evidence that the use of such additives leads to cumulative or confounding effects with the tested substance and that they need to be handled with caution. To differentiate between test substance and potential solvent effects, an adequate carrier solvent control should be integrated into the experimental design if a carrier solvent is used, although we recommend avoiding the use of carrier solvents whenever possible [46].

Due to the absence of standardized ecotoxicological test method for corals, acute toxicity tests on corals are generally associated with different test setups, exposure durations, measured endpoints and are often lacking chemical control analyses [7,8]. This makes the comparison of different studies and, thus, decision-making processes difficult or even meaningless. Although they did not establish a priori validation criteria, Conway et al. [10] conducted a study on the acute toxicity of BP-3 to the coral *Galaxea fascicularis* using a test setup that adhered to existing OECD/EPA guidelines and was similar in design to this study. They found no significant change in Fv/Fm and bleaching before the onset of mortality in *G. fascicularis*. Similar effects were observed for *M. digitata* in this study. The effects of DCMU on corals and their symbionts are well researched. Strong photoinhibition of zooxanthellae and coral bleaching are common effects that are associated with DCMU exposure [25,51,52,53]. Our results agree with these observations as strong Fv/Fm loss and bleaching were observed after 96 h of exposure to the substance. Like DCMU, the toxicity of Cu^2+^ to corals is relatively well understood, which makes it a well-suited positive control substance. The observed effects of strong bleaching followed by mortality with no significant decline of Fv/Fm are common responses of corals to Cu^2+^ exposure [21,54]. Jones [54] demonstrated that the coral host shows a stress reaction at lower Cu^2+^ concentrations than the zooxanthellae, which suggests that the host releases the symbiotic algae due to chemical stress rather than photochemical stress. Many of the cited studies derived different EC_50_/LC_50_ effect concentrations for the same chemical substances and endpoints than this study. Given that the sensitivity of different coral species to the same stressor can vary considerably [55] and results obtained from different test methods might vary markedly [8], differences in the estimated effect concentrations must be expected.

## 5. Conclusions

The standardization of a coral acute toxicity testing method in the form of an ISO/OECD test guideline is imperative to produce comparable and reliable data for use in evidence-based decision making in regulatory processes. The test method provided here may be suitable for further validation efforts as it consistently fulfilled validation criteria, facilitated the unambiguous assessment of endpoints and allowed the computation of reliable EC_50_/LC_50_ estimates. To ensure the appropriateness of the test method for standardization, validation efforts should encompass the replication of results by independent parties. Further, evaluating the ecological significance of the estimated effect concentrations requires assessing the relative sensitivity of *M. digitata* through the application of the same test method to other coral species. For a comprehensive grasp of substance toxicity across diverse exposure scenarios and life stages, forthcoming directives entail the integration of relevant (sub-)lethal endpoints into a standardizable chronic test method for adult corals and the specification of a standardizable test method for coral larvae. 

## Figures and Tables

**Table 1:** Validation criteria for water parameters and endpoint shifts.

Validation Criteria	Target
Temperature	24–28 °C, but constant within ±1.5 °C of cultivation temperature
Salinity	33–36 PSU
pH	7.7–8.5
O_2_ saturation	≥75% *
Alkalinity	5–9 °dKH
Bleaching intensity of control	≤30%
Fv/Fm of control	Fv/Fm ≥ 0.450
Tissue loss of control	≤20% (1 fragment)
Mortality of control	0%

* O_2_ saturation may be lower in replicates with mortality.

**Table 2:** Analytical results of test concentrations (*n* = 6), showing mean measured concentrations of each test substance (fresh, aged) within the 96 h semi-static test method, including their recoveries. (n.d. = no data; LoD = limit of detection).

Chem.	Nominal Conc.	Mean Measured Conc. of t_0_ to t_96_	Recovery of Nominal	Mean Measured Conc. 1st Phase		Mean Measured Conc. 2nd Phase		Recovery t_0_–t_48_	Recovery t_48_–t_96_	Mean Recovery
				t_0_	t_48_	t_48_	t_96_	Semi -static test method		
				fresh	aged	fresh	aged			
	mg L^−1^	mg L^−1^	%	mg L^−1^				%	%	%
Control	0	<LoD	100.0	<LoD	<LoD	<LoD	<LoD	100.0	100.0	100.0
BP-3	7.000	3.08700	44.1	3.37900	2.64100	3.91800	2.97300	78.2	75.9	77.1
	8.000	4.29000	53.6	4.65600	3.70200	5.32800	4.17500	79.5	78.4	79.0
	9.000	5.43400	60.4	6.13600	4.62100	6.56200	5.33300	75.3	81.3	78.3
	10.000	6.38600	63.9	7.12000	5.37200	7.81600	6.31800	75.4	80.8	78.1
	11.000	7.33900	66.7	8.10800	5.92800	9.45100	7.31000	73.1	77.3	75.2
	12.000	9.07800	75.6	10.04900	7.39500	10.54600	9.54300	73.6	90.5	82.0
Control	0	0	100.0	0	0	0	0	100.0	100.0	100.0
	0.00013	0.00018	139.1	0.00016	0.00018	0.00018	0.00017	111.5	93.0	102.2
	0.00064	0.00088	137.0	0.00081	0.00089	0.00085	0.00091	110.2	106.6	108.4
	0.00320	0.00441	137.7	0.00410	0.00442	0.00446	0.00452	107.6	101.3	104.4
	0.01600	0.01281	80.1	0.01150	0.00995	0.01607	0.01470	86.5	91.5	89.0
DCMU	0.08000	0.04937	61.7	0.04125	0.03875	0.05913	0.05918	93.9	100.1	97.0
	0.40000	0.28860	72.2	0.22682	0.21678	0.36728	0.35198	95.6	95.8	95.7
	2.00000	1.91247	95.6	1.53252	1.53191	2.28243	2.29802	100.0	100.7	100.3
	10.00000	9.39022	93.9	7.06999	7.16035	11.55859	11.69601	101.3	101.2	101.2
	50.00000	40.93958	81.9	37.01764	34.42754	50.58926	44.58775	93.0	88.1	90.6
Control	0	0.00125	100.1	0	0	0	0.005	100.0	100.0	100.0
Cu^2+^	0.005	0.00200	40.0	n.d.	0	0.003	0.003	n.d.	100.0	100.0
	0.009	0.00433	48.1	n.d.	0.003	0.005	0.005	n.d.	100.0	100.0
	0.019	0.00675	35.5	0.006	0.006	0.007	0.008	100.0	114.3	107.1
	0.038	0.01425	37.5	0.009	0.016	0.018	0.014	177.8	77.8	127.8
	0.075	0.01700	22.7	0.015	n.d.	n.d.	0.019	n.d.	n.d.	n.d.
	0.151	0.03150	20.9	0.023	0.023	0.042	0.038	100.0	90.5	95.2
	0.303	n.d.	n.d.	n.d.	n.d.	n.d.	n.d.	n.d.	n.d.	n.d.

**Table 3:** Overview of EC_50_ and LC_50_ concentrations for the tested substances and endpoints. All concentrations are given in mg L^−1^. EC_50_/LC_50_ calculations for BP-3 and DCMU are based on measured concentrations, and EC_50_/LC_50_ calculations for Cu^2+^ and DMF are based on nominal concentrations. Confidence intervals (95%) are displayed in parentheses where available.

Endpoint		Cu^2+^	BP-3	DCMU	DMF
Mortality	LC_50_	0.161 (0.055–0.267)	5.716 (5.256–6.176)	>9.390	>1416
Tissue Loss	EC_50_	0.107 (0.076–0.138)	5.112 (4.604–5.619)	>9.390	>1416
Bleaching	EC_50_	0.008 (0.005–0.011)	>9.078	0.0275 (0.01750–0.03751)	>1416
Fv/Fm Loss	EC_50_	>0.303	>9.078	0.0242 (0.01972–0.02872)	>1416
Filament Ejection	EC_50_	>0.303	>9.078	>40.939	60.055 (6.46–113.65)
Polyp Debilitation	EC_50_	>0.303	<3.087	0.0258 (−0.00013–0.05176)	>1416

## Data Availability

The data presented in this study are available on request from the corresponding authors.

## Supplementary Materials

The following supporting information can be downloaded at: https://www.mdpi.com/article/10.3390/toxics12010001/s1, Supplementary Material S1, **containing S1.1**: Coral culturing conditions; **S1.2**: PAM Fiberoptic Attachment(**Figure S1.** Construction of fiberoptic attachment for the WALZ MINI-PAM-II (Heinz Walz GmbH, Effeltrich, Germany)); **S1.3**: Morphological/Behavioral Abnormalities(**Figure S2.** Example of (A) the ejection of mesentery filaments and (B) deeply retracted (debilitated) polyps with open mouths in *Montipora digitata*, as indicated by arrows); **S1.4**: Light Curves(**Figure S3.** Light spectrum and intensity of the Radion G6 XR15 and XR30 LED lights (EcoTech LLC, Bethlehem, PA, USA), measured using a PARwise Quantum Sensor (ITC Reefculture—Seneye LTD, Norwich, England)); **S1.5**: Water Parameters(**Table S1.** Mean temperature condition during 96 h exposure of the substances BP-3, DCMU, Cu^2+^ and DMF; **Table S2**. Summary of water parameters for 96 h test periods of the substances BP-3, DCMU, Cu^2+^ and DMF); **S1.6**: Water Control Analytics (Extraction method, device settings, calibration curves, statistics) for BP-3 and DCMU(**Table S3.** Method validation of the extraction method for the substances BP-3, BP3-*d_5_*, DCMU and DCMU-*d_6_*, in respect of quality control (QC) analysis; **Table S4**. Extraction efficiency of the extraction method using internal standard recovery validation; **Figure S4**. Graph of the linear regression for the substance BP-3, displaying its coefficient of determination (R^2^) and equation; **Table S5.** Statistical analysis of the regression curve for the substance BP-3, in respect of multi-injection relative standard deviation (%RSD); **Figure S5.** Graph of the linear regression for the substance DCMU, displaying its coefficient of determination (R^2^) and equation; **Table S6.** Statistical analysis of the regression curve for the substance DCMU, in respect of multi-injection relative standard deviation (%RSD); **Table S7.** Calculation of limit of detection (LOD) and limit of quantification (LOQ) of the regression curve for the substances BP-3 and DCMU; **Table S8.** Determination of relative BP-3 carryover for the calibration curve using solvent blank post-injection; **Table S9.** Blank analysis and determination of carryover proportion in extraction blanks; **Table S10.** Calculation of estimated carryover in extracts; **S1.7**: Additional Cu^2+^ Results(**Figure S6.** Proportion of *M. digitata* fragments exposed to Cu^2+^ showing (A) mortality, (B) tissue loss, (E) ejection of mesenterial filaments and (F) polyp debilitation. Cumming plot of (C) bleaching and (D) Fv/Fm loss in *M. digitata* exposed to Cu^2+^); **S1.8**: Additional BP-3 Results(**Figure S7.** Proportion of *M. digitata* fragments exposed to BP-3 showing (A) mortality, (B) tissue loss, (E) ejection of mesenterial filaments and (F) polyp debilitation. Cumming plots of (C) bleaching and (D) Fv/Fm loss in *M. digitata* exposed to BP-3); **S1.9**: Additional DCMU Results(**Figure S8.** Proportion of *M. digitata* fragments exposed to DCMU showing (A) mortality, (B) tissue loss, (E) ejection of mesenterial filaments and (F) polyp debilitation. Cumming plots of (C) bleaching and (D) Fv/Fm loss in *M. digitata* exposed to DCMU); **S1.10**: Additional DMF Results(**Figure S9.** Proportion of *M. digitata* fragments exposed to DMF showing (A) mortality, (B) tissue loss, (E) ejection of mesenterial filaments and (F) polyp debilitation. Cumming plots of (C) bleaching and (D) Fv/Fm loss in *M. digitata* exposed to DMF); **S1.11**: Additional Effect Concentration Estimates(**Table S11**. Overview of acute effect concentrations for all tested substances (Cu^2+^, BP-3, DCMU, DMF) and endpoints (Mortality, Tissue Loss, Bleaching, Fv/Fm Loss, Filament Ejection, Polyp Debilitation); **Table S12.** Overview of statistical methods used to estimate effect concentration (cf. Table S11). The best fitting Dose Response Model (DRM) was chosen based on the lowest AIC for each individual substance and endpoint). The following supporting information can be downloaded at: https://github.com/drefeld/BleachingImageAnalysis (accessed on 18 December 2023), Supplementary Material S2, containing the Image Analysis Manual and ImageJ Macro.

## References

[1] Hughes T.P., Barnes M.L., Bellwood D.R., Cinner J.E., Cumming G.S., Jackson J.B.C., Kleypas J., van de Leemput I.A., Lough J.M., Morrison T.H. (2017). Coral Reefs in the Anthropocene. Nature.

[2] Eddy T.D., Lam V.W.Y., Reygondeau G., Cisneros-Montemayor A.M., Greer K., Palomares M.L.D., Bruno J.F., Ota Y., Cheung W.W.L. (2021). Global Decline in Capacity of Coral Reefs to Provide Ecosystem Services. One Earth.

[3] Hoegh-Guldberg O., Pendleton L., Kaup A. (2019). People and the Changing Nature of Coral Reefs. Reg. Stud. Mar. Sci..

[4] Woodhead A.J., Hicks C.C., Norström A.V., Williams G.J., Graham N.A.J. (2019). Coral Reef Ecosystem Services in the Anthropocene. Funct. Ecol..

[5] Álvarez-Muñoz D., Llorca M., Blasco J., Barceló D. (2016). Contaminants in the Marine Environment. Marine Ecotoxicology.

[6] Ouédraogo D.-Y., Mell H., Perceval O., Burga K., Domart-Coulon I., Hédouin L., Delaunay M., Guillaume M.M.M., Castelin M., Calvayrac C. (2023). What Are the Toxicity Thresholds of Chemical Pollutants for Tropical Reef-Building Corals? A Systematic Review. Environ. Evid..

[7] Mitchelmore C.L., Burns E.E., Conway A., Heyes A., Davies I.A. (2021). A Critical Review of Organic Ultraviolet Filter Exposure, Hazard, and Risk to Corals. Environ. Toxicol. Chem..

[8] Moeller M., Pawlowski S., Petersen-Thiery M., Miller I.B., Nietzer S., Heisel-Sure Y., Kellermann M.Y., Schupp P.J. (2021). Challenges in Current Coral Reef Protection—Possible Impacts of UV Filters Used in Sunscreens, a Critical Review. Front. Mar. Sci..

[9] Miller I.B., Pawlowski S., Kellermann M.Y., Petersen-Thiery M., Moeller M., Nietzer S., Schupp P.J. (2021). Toxic Effects of UV Filters from Sunscreens on Coral Reefs Revisited: Regulatory Aspects for “Reef Safe” Products. Environ. Sci. Eur..

[10] Conway A.J., Gonsior M., Clark C., Heyes A., Mitchelmore C.L. (2021). Acute Toxicity of the UV Filter Oxybenzone to the Coral Galaxea Fascicularis. Sci. Total Environ..

[11] Miller I.B., Moeller M., Kellermann M.Y., Nietzer S., Di Mauro V., Kamyab E., Pawlowski S., Petersen-Thiery M., Schupp P.J. (2022). Towards the Development of Standardized Bioassays for Corals: Acute Toxicity of the UV Filter Benzophenone-3 to Scleractinian Coral Larvae. Toxics.

[12] Pawlowski S., Moeller M., Miller I.B., Kellermann M.Y., Schupp P.J., Petersen-Thiery M. (2021). UV Filters Used in Sunscreens—A Lack in Current Coral Protection?. Integr. Environ. Assess. Manag..

[13] OECD (2004). Test No. 202: Daphnia Sp. Acute Immobilisation Test. OECD Guidelines for the Testing of Chemicals, Section 2.

[14] OECD (2019). Test No. 203: Fish, Acute Toxicity Test. OECD Guidelines for the Testing of Chemicals, Section 2.

[15] Burns E.E., Davies I.A. (2021). Coral Ecotoxicological Data Evaluation for the Environmental Safety Assessment of Ultraviolet Filters. Environ. Toxicol. Chem..

[16] Stubblefield W., Barron M., Bragin G., DeLorenzo M., De Jourdan B., Echols B., French-McCay D., Jackman P., Loughery J., Parkerton T. (2023). Improving the Design and Conduct of Aquatic Toxicity Studies with Oils Based on 20 Years of CROSERF Experience. Aquat. Toxicol..

[17] Brinkman D.L., Flores F., Luter H.M., Nordborg F.M., Brooks M., Parkerton T.F., Negri A.P. (2023). Sensitivity of the Indo-Pacific Coral Acropora Millepora to Aromatic Hydrocarbons. Environ. Pollut..

[18] He T., Tsui M.M.P., Tan C.J., Ng K.Y., Guo F.W., Wang L.H., Chen T.H., Fan T.Y., Lam P.K.S., Murphy M.B. (2019). Comparative Toxicities of Four Benzophenone Ultraviolet Filters to Two Life Stages of Two Coral Species. Sci. Total Environ..

[19] Turner N.R., Parkerton T.F., Renegar D.A. (2021). Toxicity of Two Representative Petroleum Hydrocarbons, Toluene and Phenanthrene, to Five Atlantic Coral Species. Mar. Pollut. Bull..

[20] Binet M.T., Reichelt-Brushett A., McKnight K., Golding L.A., Humphrey C., Stauber J.L. (2023). Adult Corals Are Uniquely More Sensitive to Manganese Than Coral Early-Life Stages. Environ. Toxicol. Chem..

[21] Baker A.C., Cunning R. (2015). Coral “Bleaching” as a Generalized Stress Response to Environmental Disturbance. Diseases of Coral.

[22] Van Oppen M.J.H., Lough J.M. (2018). Coral Bleaching.

[23] Bhagooli R., Mattan-Moorgawa S., Kaullysing D., Louis Y.D., Gopeechund A., Ramah S., Soondur M., Pilly S.S., Beesoo R., Wijayanti D.P. (2021). Chlorophyll Fluorescence—A Tool to Assess Photosynthetic Performance and Stress Photophysiology in Symbiotic Marine Invertebrates and Seaplants. Mar. Pollut. Bull..

[24] Marzonie M., Flores F., Sadoun N., Thomas M.C., Valada-Mennuni A., Kaserzon S., Mueller J.F., Negri A.P. (2021). Toxicity Thresholds of Nine Herbicides to Coral Symbionts (Symbiodiniaceae). Sci. Rep..

[25] Jones R. (2005). The Ecotoxicological Effects of Photosystem II Herbicides on Corals. Mar. Pollut. Bull..

[26] Roth M.S. (2014). The Engine of the Reef: Photobiology of the Coral-Algal Symbiosis. Front. Microbiol..

[27] Nalley E.M., Tuttle L.J., Barkman A.L., Conklin E.E., Wulstein D.M., Richmond R.H., Donahue M.J. (2021). Water Quality Thresholds for Coastal Contaminant Impacts on Corals: A Systematic Review and Meta-Analysis. Sci. Total Environ..

[28] Veron J.E.N. (2000). Montipora Digitata. Corals of the World.

[29] Stobart B. (2000). A Taxonomic Reappraisal of Montipora Digitata Based on Genetic and Morphometric Evidence. Zool. Stud..

[30] Johnson C.E., Goulet T.L. (2007). A Comparison of Photographic Analyses Used to Quantify Zooxanthella Density and Pigment Concentrations in Cnidarians. J. Exp. Mar. Biol. Ecol..

[31] Winters G., Holzman R., Blekhman A., Beer S., Loya Y. (2009). Photographic Assessment of Coral Chlorophyll Contents: Implications for Ecophysiological Studies and Coral Monitoring. J. Exp. Mar. Biol. Ecol..

[32] Akkaynak D., Treibitz T., Xiao B., Gürkan U.A., Allen J.J., Demirci U., Hanlon R.T. (2014). Use of Commercial Off-the-Shelf Digital Cameras for Scientific Data Acquisition and Scene-Specific Color Calibration. JOSA A.

[33] Rueden C.T., Schindelin J., Hiner M.C., DeZonia B.E., Walter A.E., Arena E.T., Eliceiri K.W. (2017). ImageJ2: ImageJ for the next Generation of Scientific Image Data. BMC Bioinform..

[34] Schindelin J., Arganda-Carreras I., Frise E., Kaynig V., Longair M., Pietzsch T., Preibisch S., Rueden C., Saalfeld S., Schmid B. (2012). Fiji: An Open-Source Platform for Biological-Image Analysis. Nat. Methods.

[35] Wickham H., Averick M., Bryan J., Chang W., McGowan L.D., François R., Grolemund G., Hayes A., Henry L., Hester J. (2019). Welcome to the Tidyverse. J. Open Source Softw..

[36] Ho J., Tumkaya T., Aryal S., Choi H., Claridge-Chang A. (2019). Moving beyond P Values: Data Analysis with Estimation Graphics. Nat. Methods.

[37] Hothorn T., Bretz F., Westfall P. (2008). Simultaneous Inference in General Parametric Models. Biom. J..

[38] Fox J., Weisberg S. (2019). An R Companion to Applied Regression.

[39] Zeileis A. (2004). Econometric Computing with HC and HAC Covariance Matrix Estimators. J. Stat. Softw..

[40] Zeileis A., Köll S., Graham N. (2020). Various Versatile Variances: An Object-Oriented Implementation of Clustered Covariances in R. J. Stat. Softw..

[41] Hothorn T., Hornik K., van de Wiel M.A., Zeileis A. (2008). Implementing a Class of Permutation Tests: The Coin Package. J. Stat. Softw..

[42] Holm S. (1979). A Simple Sequentially Rejective Multiple Test Procedure. Scand. J. Stat..

[43] Ritz C., Baty F., Streibig J.C., Gerhard D. (2015). Dose-Response Analysis Using R. PLoS ONE.

[44] Hirose K. (2006). Chemical Speciation of Trace Metals in Seawater: A Review. Anal. Sci..

[45] OECD (2011). Test No. 201: Freshwater Alga and Cyanobacteria, Growth Inhibition Test. OECD Guidelines for the Testing of Chemicals, Section 2.

[46] OECD (2019). Guidance Document on Aquatic Toxicity Testing of Difficult Substances and Mixtures. OECD Series on Testing and Assessment.

[47] European Chemicals Agency Brief Profile—Oxybenzone. https://echa.europa.eu/de/brief-profile/-/briefprofile/100.004.575.

[48] European Chemicals Agency Brief Profile—Diuron (ISO); 3-(3,4-Dichlorophenyl)-1,1-dimethylurea. https://echa.europa.eu/de/brief-profile/-/briefprofile/100.005.778.

[49] Lide D.R. (2010). CRC Handbook of Chemistry and Physics (CD-ROM Version 2010).

[50] Di Mauro V., Kamyab E., Kellermann M.Y., Moeller M., Nietzer S., Luetjens L.H., Pawlowski S., Petersen-Thiery M., Schupp P.J. (2023). Ecotoxicological Effects of Four Commonly Used Organic Solvents on the Scleractinian Coral Montipora Digitata. Toxics.

[51] Flores F., Marques J.A., Uthicke S., Fisher R., Patel F., Kaserzon S., Negri A.P. (2021). Combined Effects of Climate Change and the Herbicide Diuron on the Coral Acropora Millepora. Mar. Pollut. Bull..

[52] Jones R., Muller J., Haynes D., Schreiber U. (2003). Effects of Herbicides Diuron and Atrazine on Corals of the Great Barrier Reef, Australia. Mar. Ecol. Prog. Ser..

[53] Råberg S., Nyström M., Erös M., Plantman P. (2003). Impact of the Herbicides 2,4-D and Diuron on the Metabolism of the Coral Porites Cylindrica. Mar. Environ. Res..

[54] Jones R. (2004). Testing the Photoinhibition Model of Coral Bleaching Using Chemical Inhibitors. Mar. Ecol. Prog. Ser..

[55] Bielmyer G.K., Grosell M., Bhagooli R., Baker A.C., Langdon C., Gillette P., Capo T.R. (2010). Differential Effects of Copper on Three Species of Scleractinian Corals and Their Algal Symbionts (*Symbiodinium* spp.). Aquat. Toxicol..

