# Supplementary material for: Acute Toxicity Assays with Adult Coral Fragments: A Method for Standardization
Source: Toxics. 2023 Dec 19;12(1):1. doi: 10.3390/toxics12010001 (PMC10818607; doi:10.3390/toxics12010001)
Supplement: Supplementary file 1 [file toxics-12-00001-s001.zip › toxics-2743843-supplementary.pdf]

toxics

Supplementary Material S1 for:

Acute Toxicity Assays with Adult Coral Fragments: A Method for Standardization

David Brefeld [1,*,†], Valentina Di Mauro [1,†], Matthias Y. Kellermann [1], Samuel Nietzer [1], Mareen Moeller [1], Laura H. Lütjens [2], Sascha Pawlowski [2], Mechtild Petersen-Thiery [3] and Peter J. Schupp [1,4,*]

Table of Contents

Supplement S1.1: Coral-culturing conditions 2
Supplement S1.2: PAM Fiberoptic Attachment 2
Supplement S1.3: Morphological/Behavioral Abnormalities 3
Supplement S1.4: Light Curves 3
Supplement S1.5: Water Parameters 4
Supplement S1.6: Water Control Analytics (Extraction method, device settings, calibration curves, statistics) for BP-3 and DCMU 8
Supplement S1.7: Additional Cu^{2+} Results 13
Supplement S1.8: Additional BP-3 Results 14
Supplement S1.9: Additional DCMU Results 15
Supplement S1.10: Additional DMF Results 16
Supplement S1.11: Additional Effect Concentration Estimates 17
References 19

Supplement S1.1: Coral-culturing conditions

Montipora digitata colonies were acquired in 2013 from a commercial reseller and have been kept inside 200 L tanks connected to an approx. 6000 L recirculating aquaculture system since then. The temperature was held constant at 26.5 ± 1.5 °C, the salinity ranged between 34 and 36 PSU, the calcium concentration was kept in the range from 380 to 440 ppm and the alkalinity (KH) was adjusted to 7-7.5 °dKH. The pH ranged between 8 and 8.35. Nitrates (NO_{3-}) were below 10 mg L^{-1} and phosphate (PO_4^{3-}) was lower than 0.2 mg L^{-1}. The concentration of trace elements was monitored in regular intervals using ICP-OES water analysis. Water current inside the 200 L tanks was supplied by Turbelle® nanostream® 6045 pumps (Tunze® Aquarientechnik GmbH, Penzberg, Germany) set at approx. 4500 L h^{-1} and artificial lightning was delivered by Radion® G4 LED Lighting (EcoTech® LLC, Bethlehem, USA) at about 100-200 µmol m^{-2} s^{-1} (measured using a PARwise Quantum Sensor, ITC Reefculture – Seneye LTD, Norwich, England) in an alternating 12h dark/light cycle.

After fragmentation by drilling, the resulting holes in the mother colonies were usually overgrown after a couple of weeks if kept clean from sedimentation and algae growth. The quick recovery makes it possible to acquire new fragments from the same colony multiple times. This increases sustainability, as the dependence on coral colonies imported from the wild is minimal. The number of mother colonies can easily be scaled by producing more plates through asexual reproduction. Using the same clone of coral has the advantage of decreasing the heterogeneity among fragments and enhances the reproducibility of results.

Supplement S1.2: PAM Fiberoptic Attachment

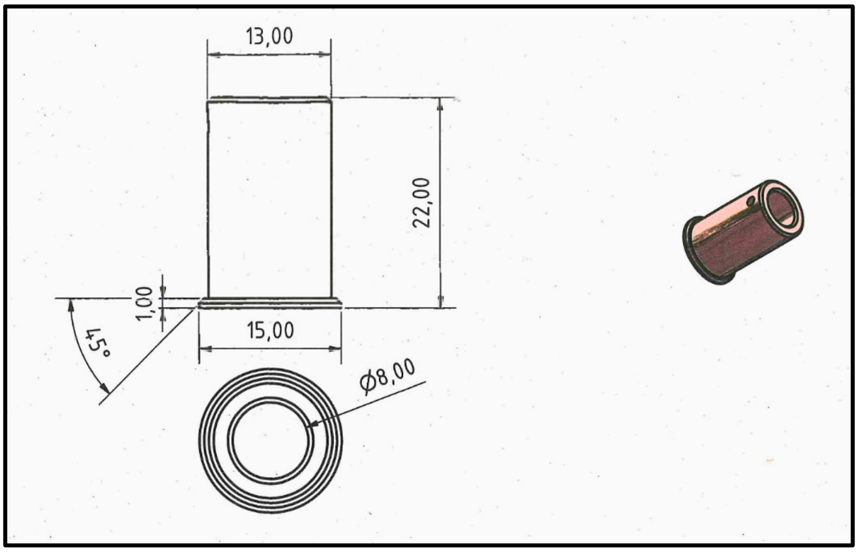

Figure S1. Construction of fiberoptic attachment for the WALZ MINI-PAM-II (Heinz Walz GmbH, Effeltrich, Germany).

Supplement S1.3: Morphological/Behavioral Abnormalities

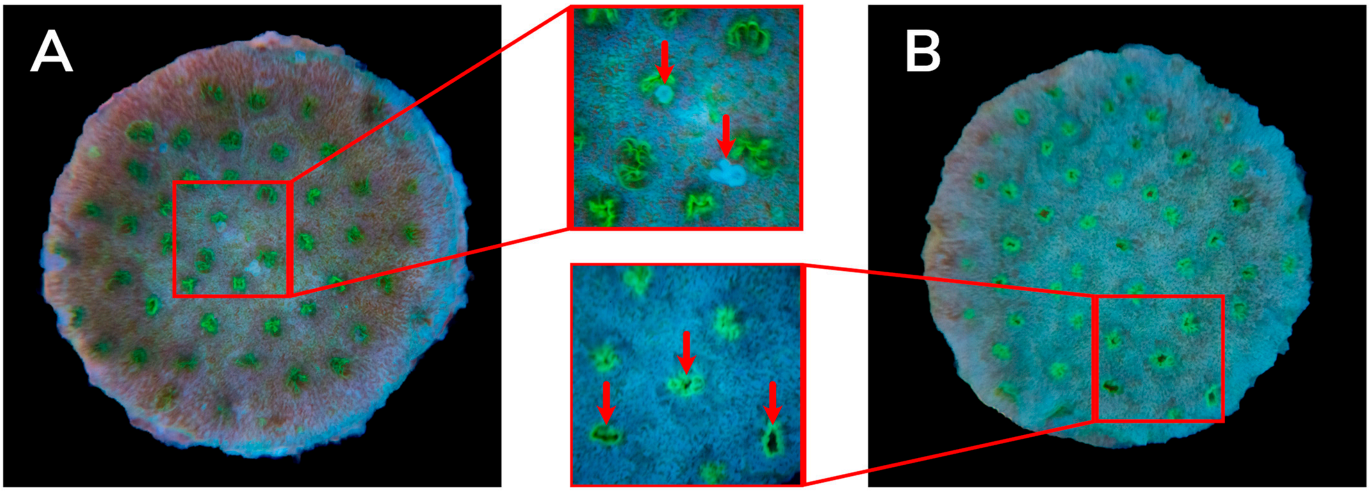

Figure S2. Example of (A) the ejection of mesentery filaments and (B) deeply retracted (debilitated) polyps with open mouths in *Montipora digitata*, as indicated by arrows.

Supplement S1.4: Light Curves

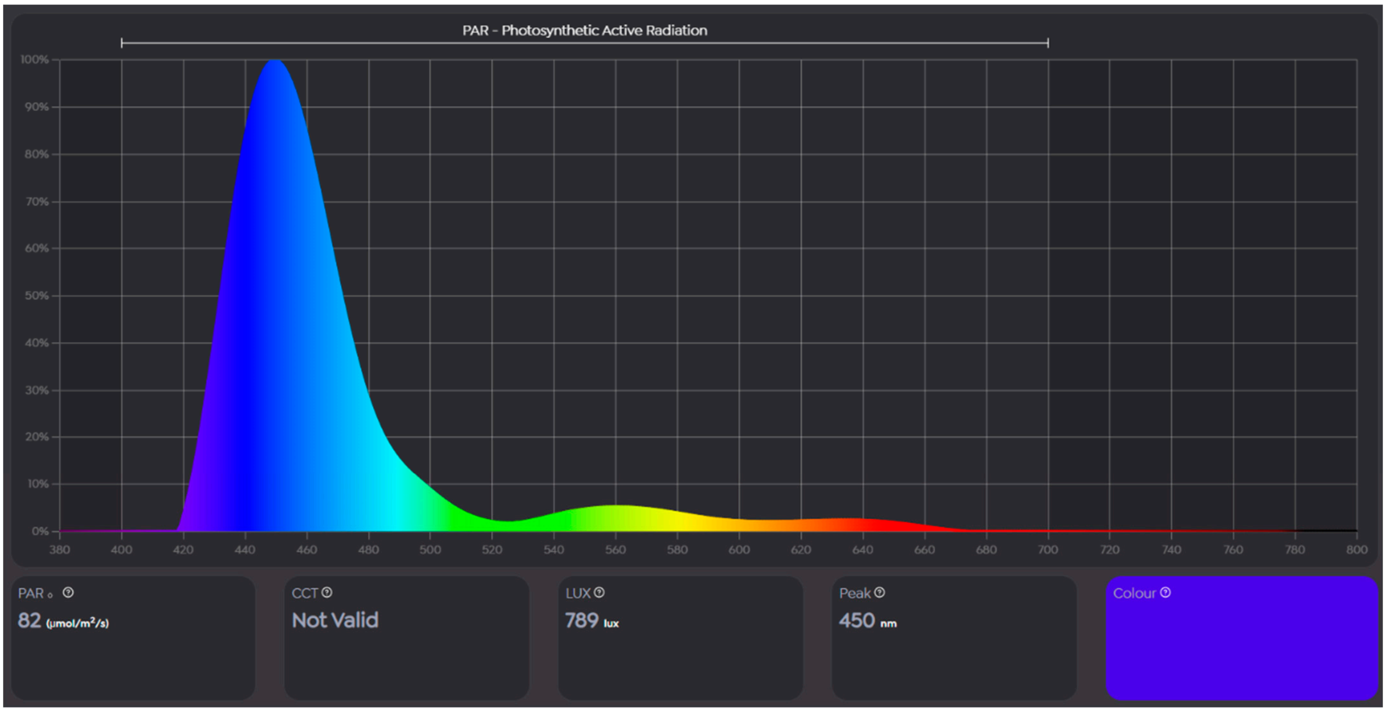

Figure S3. Light spectrum and intensity of the Radion G6 XR15 and XR30 LED lights (EcoTech LLC, Bethlehem, PA, USA), measured using a PARwise Quantum Sensor (ITC Reefculture – Seneye LTD, Norwich, England). The light spectrum and intensity on each incubator shelf were adjusted to be identical. Setting the LED lights to 0% UV, 90% violet, 90% royal blue, 90% blue, 15% green, 15% red, 30% warm white and 60% cold white light produced the shown spectra. The intensity of all wavelengths could be changed together to achieve the shown PAR intensity.

Supplement S1.5: Water Parameters

Table S1. Mean temperature condition during 96 h exposure of the substances BP-3, DCMU, Cu2+ and DMF.

Substance	*Average cultivation temperature (°C)*	*Validation criteria range (°C)*	*Mean measured exposure temperature (°C)*	*SD of mean measured exposure temperature (°C)*
BP-3	*26.50 ± 0.50*	*± 1.50*	*27.47*	*0.63*
DCMU	*26.50 ± 0.50*	*± 1.50*	*26.45*	*0.71*
Cu^{2+}	*26.50 ± 0.50*	*± 1.50*	*27.63*	*0.64*
DMF	*26.50 ± 0.50*	*± 1.50*	*27.78*	*0.48*

Table S2. Summary of water parameters for 96 h test periods of the substances BP-3, DCMU, Cu2+ and DMF.

**Compound**	**Nominal conc.**	**Mean measured conc.**	**Time**	**n**	**O_2 (%)**	**pH**	**PSU**	**KH**
BP-3	NC	NC	t_0	1	105.6	8.19	34	7.3
	7.00000	3.08700	t_0	1	106.05	8.18	33.9	7
	8.00000	4.29000	t_0	1	105.43	8.18	34.5	7.3
	9.00000	5.43400	t_0	1	105.99	8.18	34.5	7.3
	10.00000	6.38600	t_0	1	105.9	8.19	34.5	7.3
	11.00000	7.33900	t_0	1	106.28	8.18	34.5	7.45
	12.00000	9.07800	t_0	1	105.36	8.18	34.1	7.3
	PC	PC	t_0	1	105.46	8.18	34.5	7.3
	NC	NC	$t_{48\text{-aged}}$	6	96.32 ± 1.8	8.01 ± 0.04	34.82 ± 0.33	5.65 ± 0.2
	7.00000	3.08700	$t_{48\text{-aged}}$	6	94.72 ± 0.39	8.09 ± 0.02	34.97 ± 0.27	6.43 ± 0.09
	8.00000	4.29000	$t_{48\text{-aged}}$	6	94.39 ± 0.94	8.12 ± 0.01	34.95 ± 0.2	6.78 ± 0.08
	9.00000	5.43400	$t_{48\text{-aged}}$	6	92.51 ± 1.26	8.11 ± 0.01	34.87 ± 0.14	6.75 ± 0.12
	10.00000	6.38600	$t_{48\text{-aged}}$	6	90.55 ± 1.04	8.12 ± 0.01	34.87 ± 0.12	6.95 ± 0.12
	11.00000	7.33900	$t_{48\text{-aged}}$	6	82.89 ± 1.18	8.07 ± 0.01	34.92 ± 0.13	7.31 ± 0.35
	12.00000	9.07800	$t_{48\text{-aged}}$	6	59.57 ± 19.43	7.83 ± 0.19	34.92 ± 0.15	7.67 ± 0.26
	PC	PC	$t_{48\text{-aged}}$	6	96.41 ± 0.76	7.95 ± 0.06	34.98 ± 0.17	5.97 ± 0.36
	NC	NC	$t_{48\text{-fresh}}$	1	105.09	8.17	34.2	8
	7.00000	3.08700	$t_{48\text{-fresh}}$	1	105.26	8.17	34.3	7.7
	8.00000	4.29000	$t_{48\text{-fresh}}$	1	105.26	8.17	34.3	7.7
	9.00000	5.43400	$t_{48\text{-fresh}}$	1	105.42	8.17	34.3	7.85
	10.00000	6.38600	$t_{48\text{-fresh}}$	1	105.33	8.17	34.3	7.85
	11.00000	7.33900	$t_{48\text{-fresh}}$	1	106.18	8.17	34.2	7.7
	12.00000	9.07800	$t_{48\text{-fresh}}$	1	105.8	8.17	34	8
	PC	PC	$t_{48\text{-fresh}}$	1	105.87	8.17	34.4	7.7
	NC	NC	t_{96}	6	100.49 ± 0.53	7.96 ± 0.05	34.93 ± 0.27	5.98 ± 0.2

	7.00000	3.08700	t_{96}	6	91.2 ± 1.92	8.06 ± 0.01	34.95 ± 0.24	6.75 ± 0.04
	8.00000	4.29000	t_{96}	6	92.06 ± 1.07	8.11 ± 0.01	34.83 ± 0.23	7.1 ± 0.11
	9.00000	5.43400	t_{96}	6	62.19 ± 28.18	7.93 ± 0.17	34.85 ± 0.15	7.08 ± 0.13
	10.00000	6.38600	t_{96}	6	44 ± 21.83	7.79 ± 0.16	34.77 ± 0.29	7.3 ± 0.12
	11.00000	7.33900	t_{96}	6	47.02 ± 12.1	7.74 ± 0.06	34.73 ± 0.1	7.64 ± 0.33
	12.00000	9.07800	t_{96}	6	79.14 ± 8.41	8 ± 0.09	34.42 ± 0.27	8 ± 0.25
	PC	PC	t_{96}	6	96.95 ± 1.77	7.87 ± 0.06	34.92 ± 0.22	7.42 ± 0.18
DCMU	NC	NC	t_0	1	100.9	8.23	33.7	7.9
	0.00013	0.00018	t_0	1	100.2	8.23	33.9	7.8
	0.00064	0.00088	t_0	1	99.9	8.24	34	7.8
	0.00320	0.00441	t_0	1	100.2	8.24	34	7.8
	0.01600	0.01281	t_0	1	100.1	8.24	34	7.9
	0.08000	0.04937	t_0	1	100.3	8.24	34	7.8
	0.40000	0.28860	t_0	1	100.3	8.23	34	7.9
	2.00000	1.91247	t_0	1	100.3	8.24	34	7.8
	10.00000	9.39022	t_0	1	100.2	8.24	34	7.8
	50.00000	40.93958	t_0	1	100.3	8.23	34	7.8
	PC	PC	t_0	1	100.4	8.23	33.9	7.9
	NC	NC	$t_{48\text{-aged}}$	6	92.02 ± 1.56	8.05 ± 0.04	34.15 ± 0.12	6.27 ± 0.4
	0.00013	0.00018	$t_{48\text{-aged}}$	6	91.85 ± 1.4	8.04 ± 0.03	34.3 ± 0.13	6.3 ± 0.3
	0.00064	0.00088	$t_{48\text{-aged}}$	6	91.83 ± 0.63	8.04 ± 0.03	34.45 ± 0.1	6.25 ± 0.24
	0.00320	0.00441	$t_{48\text{-aged}}$	6	90.73 ± 1.12	8.05 ± 0.03	34.97 ± 1.25	6.75 ± 0.47
	0.01600	0.01281	$t_{48\text{-aged}}$	6	89.15 ± 0.73	8.06 ± 0.02	34.4 ± 0.09	6.92 ± 0.15
	0.08000	0.04937	$t_{48\text{-aged}}$	6	81.72 ± 4.38	8.08 ± 0.03	34.68 ± 0.43	7.48 ± 0.28
	0.40000	0.28860	$t_{48\text{-aged}}$	6	74.62 ± 2.96	8.05 ± 0.02	34.43 ± 0.05	7.4 ± 0.18
	2.00000	1.91247	$t_{48\text{-aged}}$	6	70.95 ± 2.59	8.02 ± 0.02	34.47 ± 0.16	7.25 ± 0.14
	10.00000	9.39022	$t_{48\text{-aged}}$	6	68.35 ± 2.01	8 ± 0.03	34.58 ± 0.17	7.48 ± 0.13
	50.00000	40.93958	$t_{48\text{-aged}}$	6	78.23 ± 5.09	8.09 ± 0.02	34.47 ± 0.1	7.58 ± 0.08
	PC	PC	$t_{48\text{-aged}}$	6	90.23 ± 2.53	8.05 ± 0.03	34.45 ± 0.1	6.52 ± 0.28
	NC	NC	$t_{48\text{-fresh}}$	1	100	8.24	34.2	7.7
	0.00013	0.00018	$t_{48\text{-fresh}}$	1	100	8.25	34.4	7.7
	0.00064	0.00088	$t_{48\text{-fresh}}$	1	99.7	8.25	34.5	7.8
	0.00320	0.00441	$t_{48\text{-fresh}}$	1	99.9	8.25	34.5	7.6
	0.01600	0.01281	$t_{48\text{-fresh}}$	1	100.1	8.25	34.6	7.9
	0.08000	0.04937	$t_{48\text{-fresh}}$	1	100.2	8.25	34.5	7.7
	0.40000	0.28860	$t_{48\text{-fresh}}$	1	100.1	8.25	34.5	7.9
	2.00000	1.91247	$t_{48\text{-fresh}}$	1	100.1	8.25	34.5	7.7
	10.00000	9.39022	$t_{48\text{-fresh}}$	1	100.3	8.25	34.5	7.9
	50.00000	40.93958	$t_{48\text{-fresh}}$	1	100.1	8.24	34	7.7
	PC	PC	$t_{48\text{-fresh}}$	1	100.1	8.24	34.3	7.8
	NC	NC	t_{96}	6	94.53 ± 0.92	8.05 ± 0.04	35.05 ± 0.87	6.48 ± 0.34
	0.00013	0.000180	t_{96}	6	93.68 ± 0.35	8 ± 0.01	34.75 ± 0.19	6.4 ± 0.21
	0.00064	0.000880	t_{96}	6	93.33 ± 0.57	8.04 ± 0.02	34.97 ± 0.14	6.48 ± 0.15
	0.00320	0.004410	t_{96}	6	94.82 ± 0.54	8.08 ± 0.03	34.97 ± 0.2	6.82 ± 0.2
	0.01600	0.012810	t_{96}	6	84.47 ± 1.53	8.09 ± 0.01	35.17 ± 0.51	7.68 ± 0.2
	0.08000	0.049370	t_{96}	6	77.13 ± 6.31	8.08 ± 0.02	35.08 ± 0.2	7.85 ± 0.12
	0.40000	0.288600	t_{96}	6	76.47 ± 7.17	8.08 ± 0.03	35.28 ± 0.89	7.75 ± 0.24
	2.00000	1.912470	t_{96}	6	70.2 ± 2.21	8.04 ± 0.03	34.95 ± 0.14	7.63 ± 0.12

	10.00000	9.390220	t_{96}	6	72.8 ± 2.49	8.04 ± 0.01	35.02 ± 0.08	7.77 ± 0.14
	50.00000	40.939580	t_{96}	6	26.33 ± 19.12	7.73 ± 0.08	35.07 ± 0.22	8.08 ± 0.08
	PC	PC	t_{96}	6	95.03 ± 0.25	8.05 ± 0.04	35.1 ± 0.14	7.65 ± 0.12
Cu^{2+}	NC	NC	t_0	1	98.18	8.21	33.8	8.15
	0.00500	0.00200	t_0	1	98.89	8.23	33.8	8
	0.00900	0.00433	t_0	1	98.99	8.22	33.8	8.15
	0.01900	0.00675	t_0	1	98.72	8.22	33.8	8.15
	0.03800	0.01425	t_0	1	98.33	8.22	33.8	8
	0.15100	0.03150	t_0	1	98.13	8.22	33.8	8
	0.30300	n.m.	t_0	1	98.82	8.22	33.8	8.15
	NC	NC	$t_{48\text{-aged}}$	12	90.72 ± 3.04	8.06 ± 0.02	34.8 ± 0.47	7.18 ± 0.19
	0.00500	0.00200	$t_{48\text{-aged}}$	6	92.02 ± 0.63	8.05 ± 0.04	34.57 ± 0.14	7 ± 0.27
	0.00900	0.00433	$t_{48\text{-aged}}$	6	91.56 ± 1.03	8.06 ± 0.03	34.6 ± 0.15	7.07 ± 0.33
	0.01900	0.00675	$t_{48\text{-aged}}$	6	91.37 ± 0.6	8.05 ± 0.04	34.7 ± 0.15	7.03 ± 0.22
	0.03800	0.01425	$t_{48\text{-aged}}$	6	95.86 ± 0.66	8.07 ± 0.05	34.82 ± 0.26	7.43 ± 0.28
	0.07600	0.01700	$t_{48\text{-aged}}$	6	93.69 ± 1.19	8.06 ± 0.02	34.82 ± 0.25	7.19 ± 0.29
	0.15100	0.03150	$t_{48\text{-aged}}$	6	92.77 ± 0.83	8.14 ± 0.02	34.83 ± 0.15	7.82 ± 0.39
	0.30300	n.m.	$t_{48\text{-aged}}$	6	93.08 ± 1.63	8.15 ± 0.02	34.8 ± 0.2	8.35 ± 0.2
	NC	NC	$t_{48\text{-fresh}}$	1	98.83	8.19	34.1	7.7
	0.00500	0.00200	$t_{48\text{-fresh}}$	1	97.56	8.2	34.1	7.85
	0.00900	0.00433	$t_{48\text{-fresh}}$	1	98.78	8.21	34.2	7.7
	0.01900	0.00675	$t_{48\text{-fresh}}$	1	99.3	8.21	34.3	8.15
	0.03800	0.01425	$t_{48\text{-fresh}}$	1	98.37	8.21	34.2	7.7
	0.15100	0.01700	$t_{48\text{-fresh}}$	1	98.79	8.21	34.2	8
	0.30300	0.03150	$t_{48\text{-fresh}}$	1	98.7	8.2	34.2	8
	NC	NC	t_{96}	12	93.03 ± 2.16	7.99 ± 0.02	35.08 ± 0.29	6.2 ± 0.55
	0.00500	0.00200	t_{96}	6	93.41 ± 0.9	7.93 ± 0.03	34.77 ± 0.08	5.52 ± 0.32
	0.00900	0.00433	t_{96}	6	93.48 ± 0.62	7.93 ± 0.04	34.83 ± 0.12	5.42 ± 0.2
	0.01900	0.00675	t_{96}	6	91.17 ± 0.57	7.92 ± 0.04	35.12 ± 0.4	5.73 ± 0.46
	0.03800	0.01425	t_{96}	6	92.77 ± 2.49	7.92 ± 0.14	34.98 ± 0.37	6.6 ± 0.18
	0.07600	0.01700	t_{96}	6	90.48 ± 0.44	7.97 ± 0.03	34.95 ± 0.24	6.53 ± 0.31
	0.15100	0.03150	t_{96}	6	90.63 ± 0.92	8.06 ± 0.03	35.02 ± 0.27	7.47 ± 0.13
	0.30300	n.m.	t_{96}	6	63.62 ± 13.24	7.92 ± 0.13	35.02 ± 0.25	8.33 ± 0.15
DMF	NC		t_0	1	95.65	8.18	33.4	8.1
	5.83000		t_0	1	96.03	8.16	33.7	7.5
	17.48000		t_0	1	96.1	8.16	33.7	7.8
	52.44000		t_0	1	95.72	8.15	33.8	8
	157.33000		t_0	1	95.48	8.16	33.8	8
	472.00000		t_0	1	95.41	8.13	33.7	7.7
	1416.00000		t_0	1	95.26	8.18	33.6	7.7
	PC		t_0	1	95.55	8.16	33.5	7.5
	NC		$t_{48\text{-aged}}$	6	91.25 ± 0.97	8.02 ± 0.05	34.45 ± 0.12	6.38 ± 0.36
	5.83000		$t_{48\text{-aged}}$	6	92.12 ± 0.46	8.01 ± 0.05	34.85 ± 0.46	6.46 ± 0.51
	17.48000		$t_{48\text{-aged}}$	6	91.84 ± 0.81	7.99 ± 0.03	34.75 ± 0.18	6.08 ± 0.22
	52.44000		$t_{48\text{-aged}}$	6	91.58 ± 0.34	8.02 ± 0.02	34.43 ± 0.21	6.08 ± 0.22
	157.33000		$t_{48\text{-aged}}$	6	90.36 ± 0.3	8 ± 0.04	34.62 ± 0.19	6.16 ± 0.36
	472.00000		$t_{48\text{-aged}}$	6	90.52 ± 0.9	8.01 ± 0.07	34.43 ± 0.4	6.59 ± 0.57
	1416.00000		$t_{48\text{-aged}}$	6	89.75 ± 0.91	8.03 ± 0.03	34.37 ± 0.23	6.8 ± 0.08

PC	$t_{48\text{-aged}}$	6	90.31 ± 1.16	8 ± 0.03	34.6 ± 0.25	6.78 ± 0.23
NC	$t_{48\text{-fresh}}$	1	97.92	8.23	33.9	7.8
5.83000	$t_{48\text{-fresh}}$	1	100.42	8.24	33.8	7.85
17.48000	$t_{48\text{-fresh}}$	1	98.58	8.23	33.8	7.85
52.44000	$t_{48\text{-fresh}}$	1	100.42	8.22	33.6	7.85
157.33000	$t_{48\text{-fresh}}$	1	98.65	8.22	33.8	7.7
472.00000	$t_{48\text{-fresh}}$	1	98.95	8.22	33.8	7.7
1416.00000	$t_{48\text{-fresh}}$	1	100.62	8.22	33.7	7.85
PC	$t_{48\text{-fresh}}$	1	100.38	8.21	33.6	7.5
NC	t_{96}	6	90.93 ± 1.25	7.97 ± 0.03	34.97 ± 0.15	5.97 ± 0.21
5.83000	t_{96}	6	89.5 ± 0.85	7.96 ± 0.04	35.12 ± 0.56	6.09 ± 0.42
17.48000	t_{96}	6	88.91 ± 0.43	7.97 ± 0.03	35.07 ± 0.65	5.93 ± 0.27
52.44000	t_{96}	6	90.11 ± 0.46	7.98 ± 0.01	34.68 ± 0.23	5.99 ± 0.17
157.33000	t_{96}	5	88.1 ± 0.54	7.97 ± 0.02	34.78 ± 0.18	5.99 ± 0.25
472.00000	t_{96}	6	86.67 ± 1.25	8.01 ± 0.03	34.9 ± 0.56	6.9 ± 0.53
1416.00000	t_{96}	6	88.15 ± 1.19	8.07 ± 0.02	34.87 ± 0.21	7.15 ± 0.16
PC	t_{96}	6	87.09 ± 0.81	7.97 ± 0.04	34.72 ± 0.16	6.38 ± 0.24

Supplement S1.6: Water Control Analytics (Extraction method, device settings, calibration curves, statistics) for BP-3 and DCMU

Detailed extraction method and UPLC-MS Analyses for BP-3 and DCMU

The extraction method was adapted from Miller et al. 2022 [1] and modified for the extraction of organic compounds dissolved in seawater media.

The 5 mL water samples, stored at -20°C, were gently defrosted and further acidified with 7 µL formic acid (FA; Biosolve Chimie SARL, ULC-MS grade, 99% purity, Dieuze, France, CAS 64-18-6) to reach a pH of ≤ 4. The extraction was performed with 100 µL of the highly lipophilic organic extractant tetrachloroethylene (PCE; Honeywell Specialty Chemicals Seelze GmbH, HPLC grade, ≥99.9% purity, Seelze, Germany, CAS 127-18-4) followed by a constant and vigorous 2 min of vortexing (RS-VA 10, Phoenix Instruments GmbH, Garbsen, Germany) at 2500 rpm and an additional centrifugation time of 10 min at 1750 rpm (Sigma 3-16KL; Sigma Laborzentrifugen GmbH, Osterode am Harz, Germany). For DCMU, a disperser solvent, namely, ethyl acetate (EtOAc; VWR International bvba, HPLC grade, ≥99.8% purity, Leuven, Belgium, CAS 141-78-6) in a 1:1 ratio with PCE, had to be added to increase its extraction efficiency. We aimed to achieve a 50-fold BP-3 and DCMU concentration in the resulting droplet of PCE at the bottom of the glass vial after the procedure. For validation of the extraction efficiency, an internal standard (IS; deuteride BP-3 as BP3-d_5 and deuteride DCMU as DCMU-d_6 (Sigma-Aldrich Co., analytical standard, St. Louis, US, CAS 1219798-54-5, and CAS 1007536-67-5, respectively)) with a final analytical concentration of 1 or 0.5 µg L-1 was applied to the water samples before PCE extraction. For measuring the BP-3 concentrations, 5 µL of the formed PCE droplet was transferred into 2 mL amber glass vials (neochrome ND9, neoLab Migge GmbH, Heidelberg, Germany) and speed-vacuumed (Sigma 3-16KL; Sigma Laborzentrifugen GmbH, Osterode am Harz, Germany) for 21 min at -4.6 bar for a 100% PCE reduction. After PCE reduction, the amber glass vials rested for another 30 min (lids open) under the fumehood before refilling with 1 mL of methanol (MeOH; Biosolve Chimie SARL, ULC-MS grade, >99.98% purity, Dieuze, France, CAS 67-56-1) and diluting 10-fold to get into the analytical range of the instrument. Almost the same procedure was used for DCMU, varying only the amount of droplet transferred as the test concentrations ranged more widely. To quantify the BP-3 and DCMU test concentrations, an external (solvent) linear calibration curve made from an inhouse BP-3 and DCMU standard (100% diluted in MeOH) was used, with a five-step dilution series (i.e., 400, 200, 100, 50, 25 (µg L-1)) for BP-3 and an 11-step dilution series (i.e., 500, 100, 50, 10, 5, 1, 0.5, 0.1, 0.05, 0.01, 0.005 (µg L-1)) for DCMU (Supplement S1.6.3). For validation, each calibration point and sample contained a deuterated IS with a concentration of 5, 1 or 0.5 µg L-1, respectively. Performing a calibration and method validation, quality controls (QCs; n ≥ 3) for all target compounds (i.e., BP-3, BP3-d_5, DCMU and DCMU-d_6) were included and highlighted %RSD < 3 with R2 > 0.99 for the calibration curve and %RSD < 6 for the extraction method with a valid mean recovery of 105% (BP3-d_5) and 57 % (DCMU-d_6) (cf. Supplement S1.6.2). Carryover of 0.0005 – 0.0008 mg L-1 for BP-3 during analysis did not affect the analytical results while being under the limit of detection (LOD = 0.02 mg L-1) and the limit of quantification (LOQ= 0.07 mg L-1) of the calibration curve (cf. Supplement S1.6.4 and S1.6.5). For DCMU, the calibration curve resulted in LOD = 0.0006 mg L-1 and LOQ = 0.0017 mg L-1 (cf. Supplement S1.6.4).

Chromatographic analyses of the extract samples were performed by WATERS ACQUITY H Class Plus ultraperformance liquid chromatography (UPLC) connected to micro mass spectrometer (µMS) (Waters Xevo TQ-S micro, Waters Co., Milford, MA, USA). The total run time of the UPLC-MS system was 7 minutes for BP-3 and 8 minutes for DCMU. During measurement, samples were held at 10 °C in a Waters Sample Manager with Flow-Through Needle (SM-FTN). Using IPA-MeOH (70:30) as wash and MeOH-H_2O (50:50) as purge solution, needle and syringe were rinsed with a 3 sec pre-inject and 6 sec post-inject wash between measurements. Then, 1 µL of a sample was injected into the UPLC-MS system by passing an ACQUITY UPLC BEH C_{18} column (1.7 µm; 2.1 x 150 mm) that was

heated prior and kept at 40 °C. A gradient of C (85% MeOH, 15% H_2O, 0.1% ammonium hydroxide (NH_4OH), 0.04% formic acid (FA)) and D (50% MeOH, 50% isopropanol (IPA), 0.1% NH_4OH, 0.04% FA) with a flow rate of 0.2 mL min^{-1} was regulated by a Waters ACQUITY quaternary solvent manger to ensure a gradient elution of the target compound.

After passing the UPLC column, the compounds were directed into the MS system through an electron spray ionizing source set in positive mode (ES^+). The method condition of the MS system was adjusted using Waters instruments integrated software IntelliStart performing multiple reaction monitoring (MRM). During measurement, the source temperature was kept at 550 °C with an average capillary voltage of 2 kV and an average cone voltage of 20 V. The source was held at a desolvation gas flow rate of 1000 L h^{-1} and a cone gas flow rate of 50 L h^{-1}. With the MRM performance, the parent ion of BP-3 (229.075 *m*/*z*) was identified using a quantification ion (151.0097 *m*/*z*) and a first target ion (104.9450 *m*/*z*). For the deuterated internal standard (IS) of BP-3, the parent ion of BP-3-d_5 (234.1386 *m*/*z*) was detected by the quantification ion (151.1342 *m*/*z*) and the first target ion (109.8897 *m*/*z*). In respect of DCMU, the parent ion (232.9040 *m*/*z*) was identified using a quantification ion (71.8930 *m*/*z*) and a first target ion (45.9460 *m*/*z*). For the deuterated IS of DCMU, the parent ion of DCMU-d_6 (238.9040 *m*/*z*) was detected by the quantification ion (78.0070 *m*/*z*) and the first target ion (51.9310 *m*/*z*).

Table S3. Method validation of the extraction method for the substances BP-3, BP3-d_5, DCMU and DCMU-d_6, in respect of quality control (QC) analysis.

Compound	Conc. (µg L^{-1})	Analytical conc. (µg L^{-1})	Conc. IS (µg L^{-1})	n	Mean (PA)	SD (PA)	%RSD
BP-3	6000	150	0.5	16	1552593	65782.4	4.24
BP3-d_5 (IS)	2.0	0.5		16	2659	112.7	4.24
DCMU	5000	125	5	6	1822888	44845.1	2.46
	50.0	12.5	0.5	6	170210	4291.4	2.52
	0.5	0.625	0.5	6	7917	461.5	5.83
DCMU-d_6 (IS)	200	5		6	74487.5	1636.4	2.20
	2.0	0.5		6	7356.3	165.4	2.25
	0.4	0.5		6	7343.7	137.8	1.88

Table S4. Extraction efficiency of the extraction method using internal standard recovery validation.

				Recovery		
**Compound**	**n**	**min**	**max**	**Mean**	**SD**	**SE**
				**%**		
**BP3-*d*5 (IS)**	**16**	95	112	**105**	4.42	1.11
**DCMU-*d*6 (IS)**	**18**	54	60	**57**	1.45	0.27

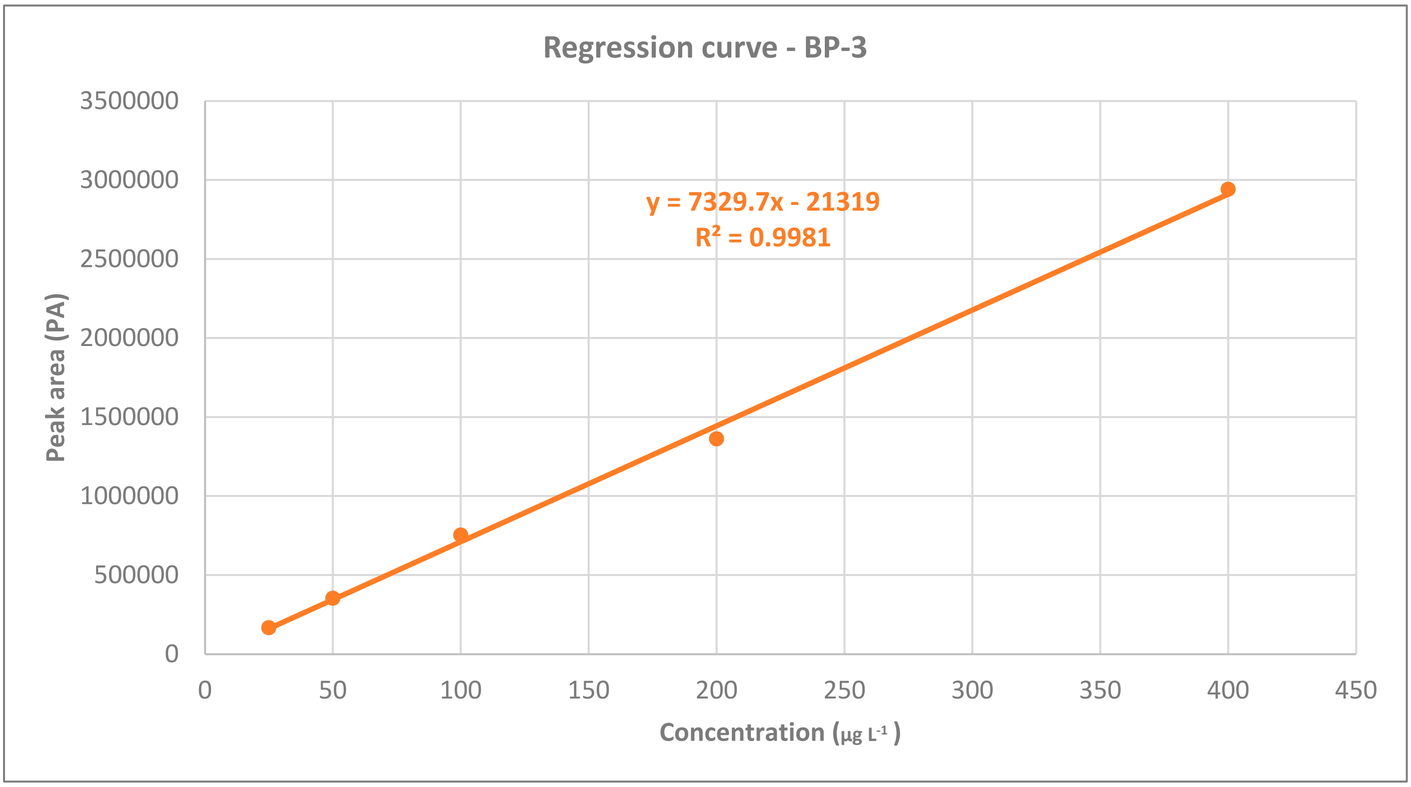

Figure S4. Graph of the linear regression for the substance BP-3, displaying its coefficient of determination (R^2) and equation.

Table S5. Statistical analysis of the regression curve for the substance BP-3, in respect of multi-injection relative standard deviation (%RSD).

Multi-injection (quality control—QC)	Concentration (µg L^{-1})	n	Mean (PA)	SD (PA)	%RSD
	25	3	166690	1485.8	0.9
	50	3	352785	2090.2	0.6
	100	3	752440	3033.7	0.4
	200	3	1361146	7310.1	0.5

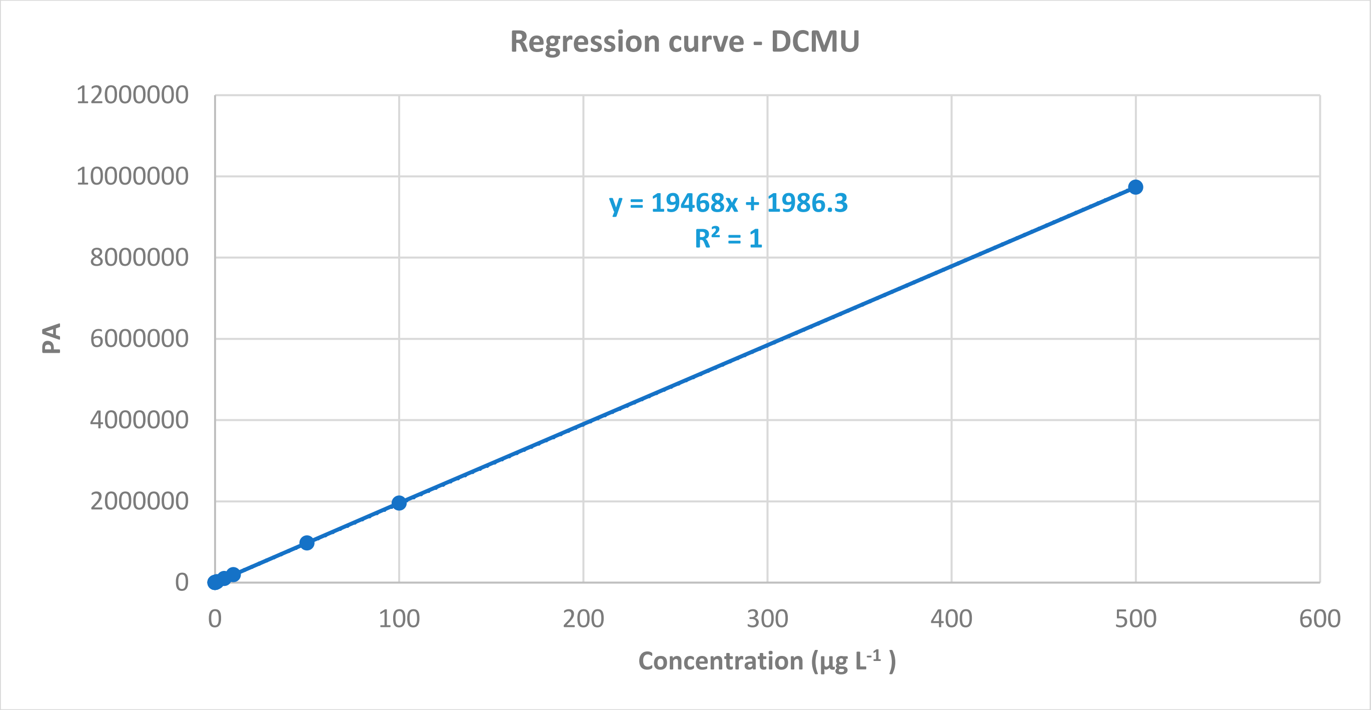

Figure S5. Graph of the linear regression for the substance DCMU, displaying its coefficient of determination (R^2) and equation.

Table S6. Statistical analysis of the regression curve for the substance DCMU, in respect of multi-injection relative standard deviation (%RSD).

Multi-injection (quality control—QC)	Concentration (µg/L)	n	Mean (PA)	SD (PA)	%RSD
	0.01	4	548	9.2	1.7
	0.10	4	2332	51.8	2.2
	10.00	4	195326	1162.6	0.6
	100.00	4	1942245	13174.7	0.7

Table S7. Calculation of limit of detection (LOD) and limit of quantification (LOQ) of the regression curve for the substances BP-3 and DCMU.

**Compound**	**BP-3**	**DCMU**
Unit	µg L^{-1}	µg L^{-1}
Equation of regression curve	*y = 7329.7x - 21319*	*y = 19468x + 1986.3*
Conc. range of regression curve	25 - 400	0.005 - 500
Calculation formulas	*LOD = (3.3*δ)/s; LOQ = (10*δ)/s*	
δ (SD residuals)	49063.2	3392.1
s (intercept)	7329.7	19468.4
LOD	22.1	0.6
LOQ	66.9	1.7

Table S8. Determination of relative BP-3 carryover for the calibration curve using solvent blank post-injection.

Nominal conc. (µg L^{-1})	*25*	*Blank*	*50*	*Blank*	*100*	*Blank*	*400*	Blank
Measured peak areas (PAs)	160265	398	346568	425	740562	472	2899698	816
	170181	163	350917	546	749859	233	2910696	422
	175249	400	366116	183	773318	360	3012094	431
Average	168565	320	354534	385	754580	355	2940829	556
SD	7622	136	10264	185	16881	120	61962	225
RC (%) *	0.190		0.108		0.047		0.019	

*Relative carryover (RC) expressed as ((Average blank PA/Average conc. PA) x 100).

Table S9. Blank analysis and determination of carryover proportion in extraction blanks.

Type	n	Mean (PA)	SD	SE
Extraction blank	18	45	85.7	20.2
Solvent blank	88	409	235.5	25.1
%CEB*		11		

*Proportion of carryover in extraction blank (%CEB) expressed as ((Mean extraction blank PA / Mean solvent blank PA) x 100).

Table S10. Calculation of estimated carryover in extracts.

Nominal conc. (µg L^{-1})	*25*	*50*	*100*	*400*
Measured conc. (µg L^{-1})	25.220	51.030	105.615	404.744
	25.045	50.204	105.738	406.533
	25.007	50.663	105.397	406.322
Mean	25.091	50.632	105.583	405.866
SD	0.114	0.414	0.173	0.978
%RSD	0.453	0.817	0.164	0.241
Accuracy (%)	100.363	101.265	105.583	101.467
Absolute error (µg L^{-1})	0.091	0.632	5.583	5.866
RC (%)	0.190	0.108	0.047	0.019
EC* (µg L^{-1})	4.768	5.494	4.967	7.678
EC (mg L^{-1})	0.005	0.005	0.005	0.008
%CEB	11	11	11	11
CEB (calculation factor)	0.11	0.11	0.11	0.11
ECE** (mg L^{-1})	0.0005	0.0006	0.0005	0.0008

*Estimated carryover (EC) concentration expressed as (RC (%) x Mean measured conc.); ** Estimated carryover in extracts (ECE) expressed as (EC x CEB).

Supplement S1.7: Additional Cu^{2+} Results

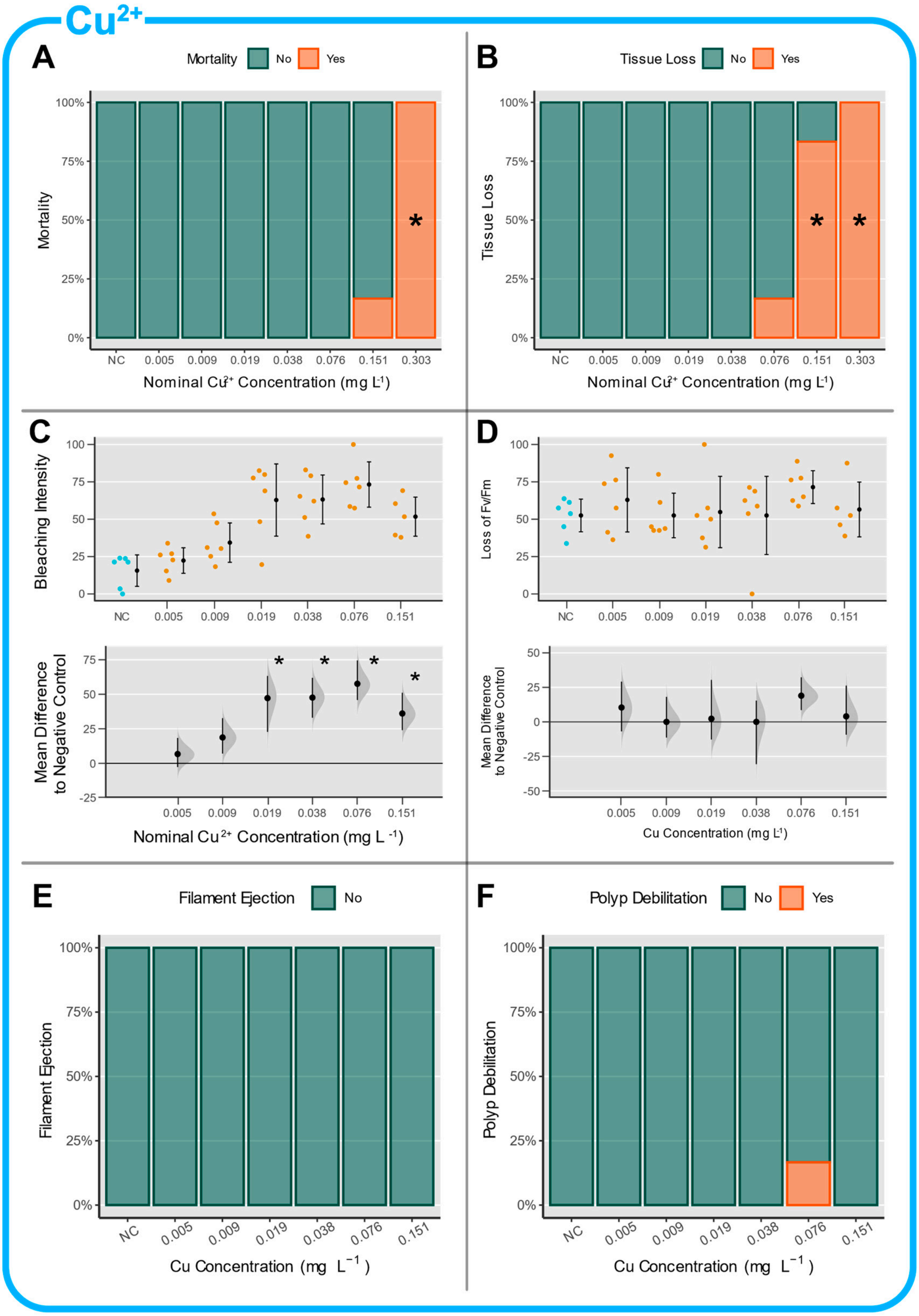

Figure S6. Proportion of *M. digitata* fragments exposed to Cu^{2+} showing (A) mortality, (B) tissue loss, (E) ejection of mesenterial filaments and (F) polyp debilitation. Cumming plot of (C) bleaching and (D) Fv/Fm loss in *M. digitata* exposed to Cu^{2+}, showing the datapoints with mean and SD, and the mean difference of each concentration to the negative control (NC) with bootstrap 95% CIs. Asterisks indicate statistically significant differences to NC.

Supplement S1.8: Additional BP-3 Results

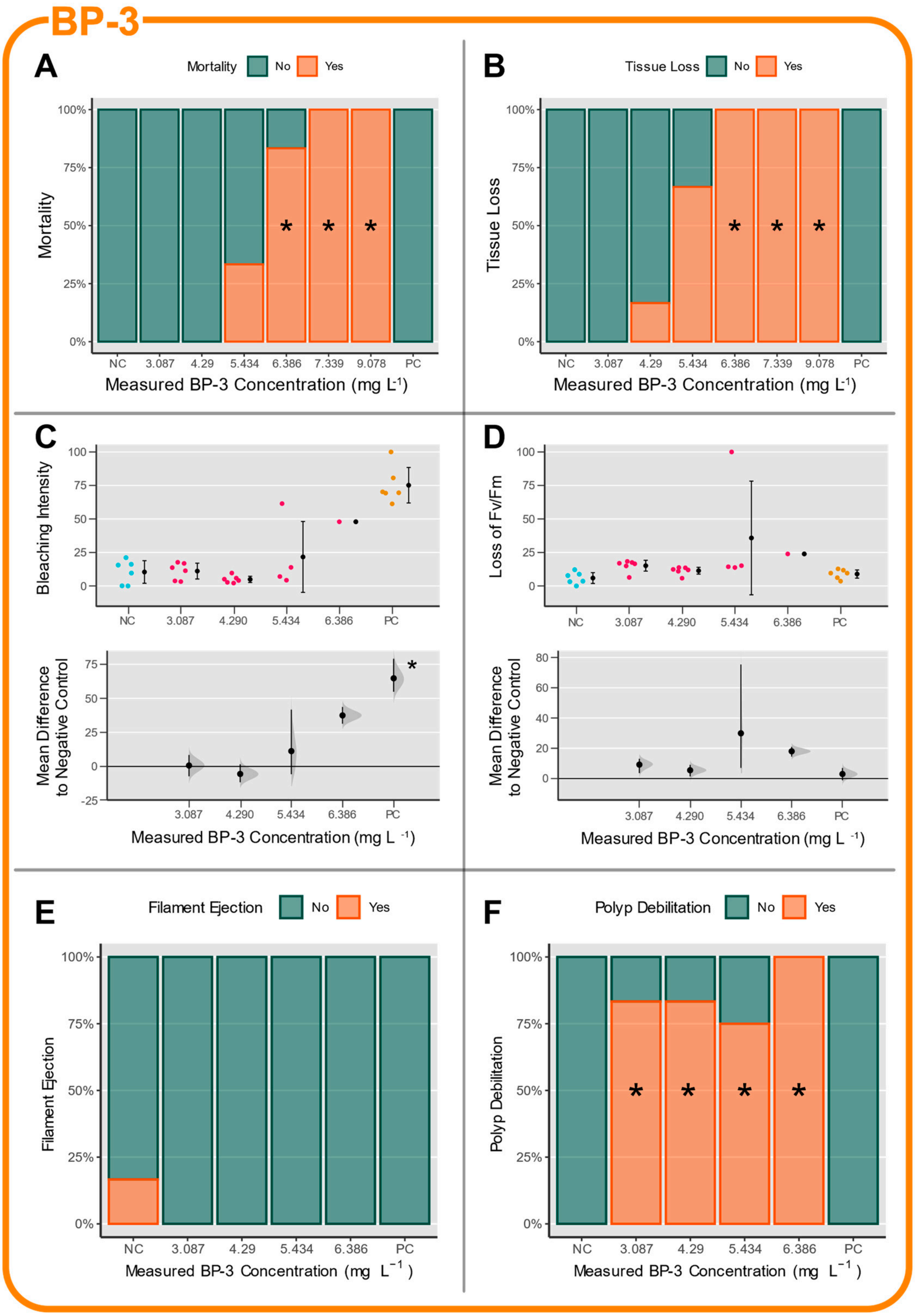

Figure S7. Proportion of *M. digitata* fragments exposed to BP-3 showing (A) mortality, (B) tissue loss, (E) ejection of mesenterial filaments and (F) polyp debilitation. Cumming plots of (C) bleaching and (D) Fv/Fm loss in *M. digitata* exposed to BP-3, showing the datapoints with mean and SD, and the mean difference of each concentration to the negative control (NC) with bootstrap 95% CIs. Asterisks indicate statistically significant differences to NC. "PC" indicates the positive control (0.08 mg L^{-1} $CuCl_2$).

Supplement S1.9: Additional DCMU Results

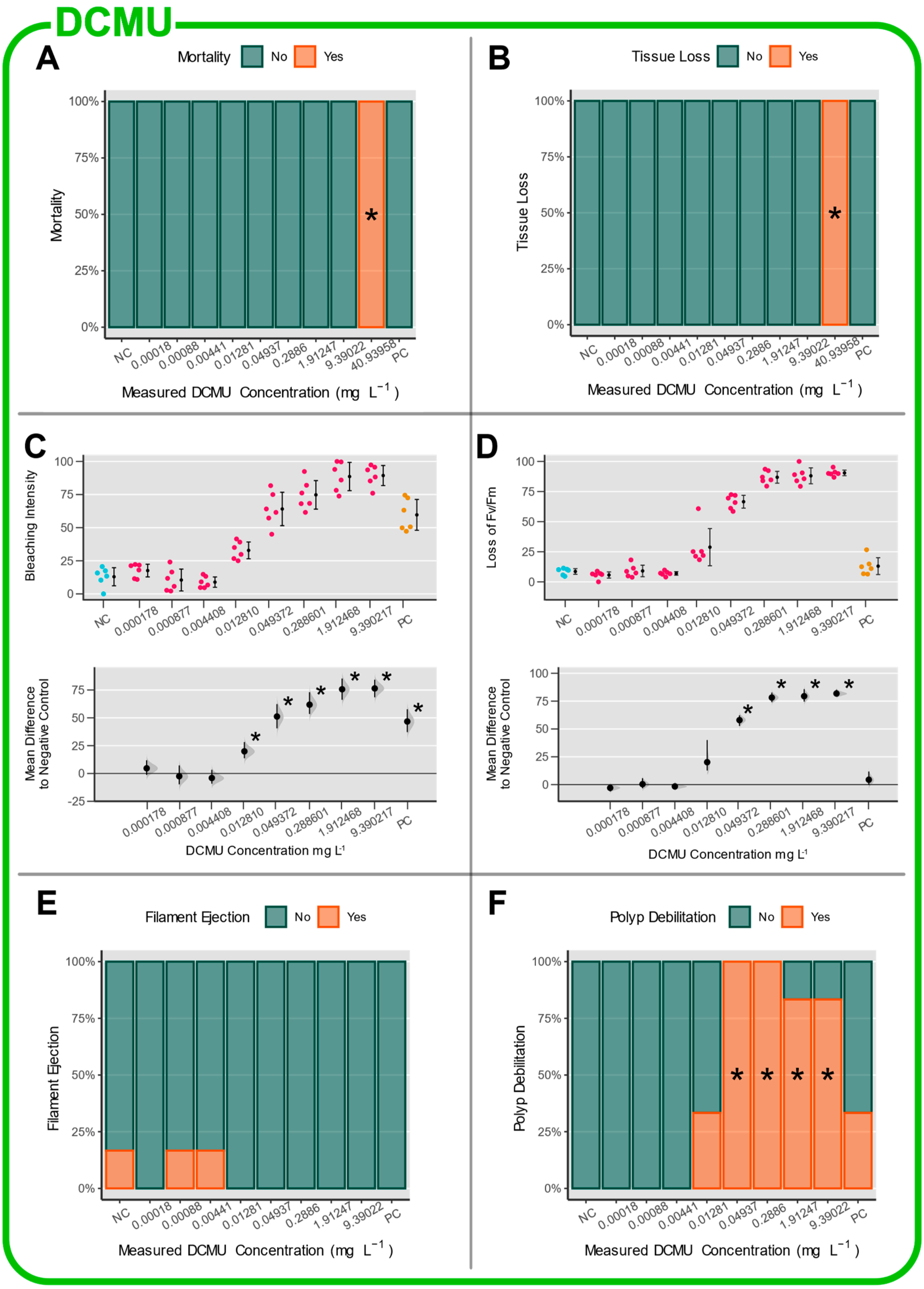

Figure S8. Proportion of *M. digitata* fragments exposed to DCMU showing (A) mortality, (B) tissue loss, (E) ejection of mesenterial filaments and (F) polyp debilitation. Cumming plots of (C) bleaching and (D) Fv/Fm loss in *M. digitata* exposed to DCMU, showing the datapoints with mean and SD, and the mean difference of each concentration to the negative control (NC) with bootstrap 95% CIs. Asterisks indicate statistically significant differences to NC. "PC" indicates the positive control (0.08 mg L^{-1} $CuCl_2$).

Supplement S1.10: Additional DMF Results

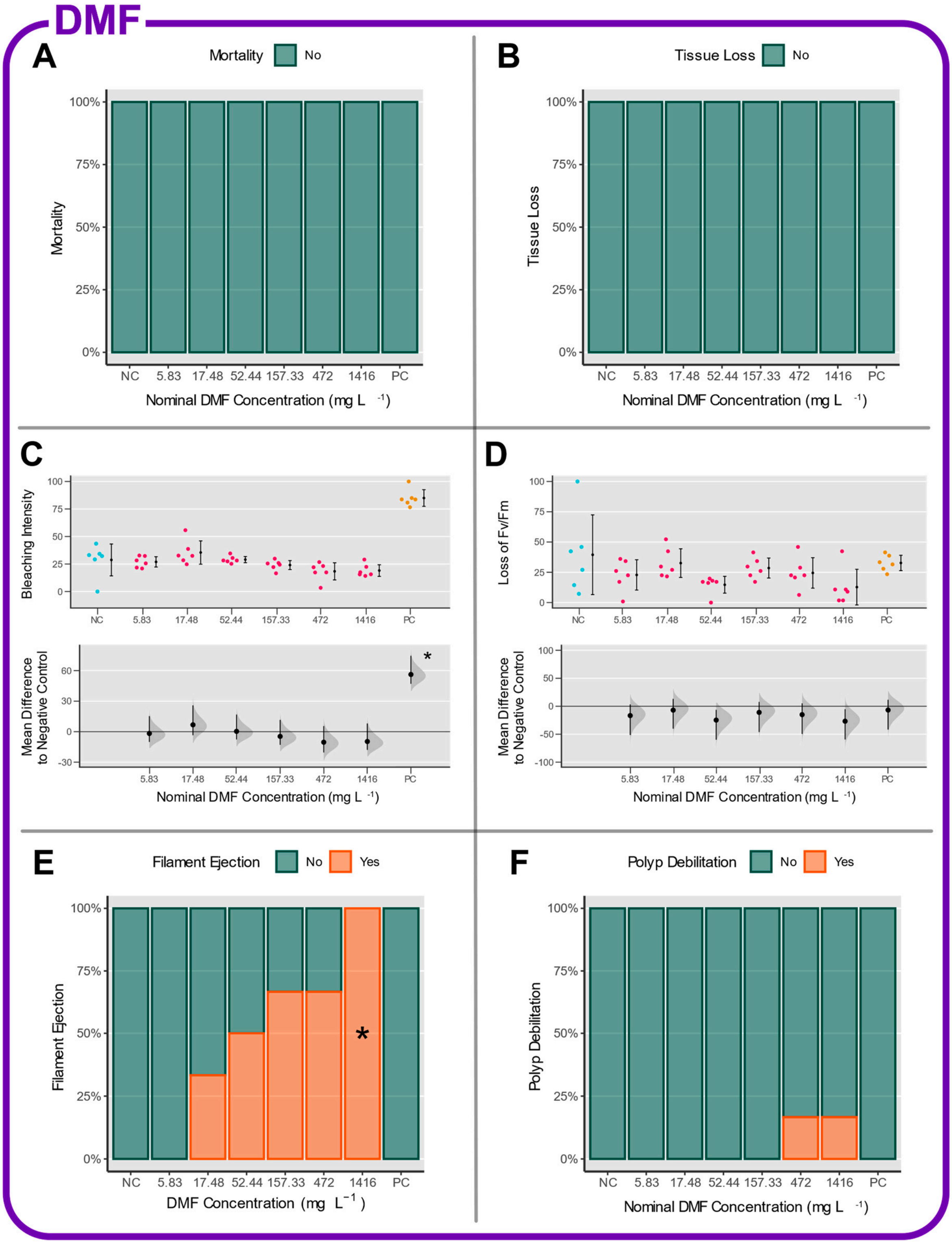

Figure S9. Proportion of *M. digitata* fragments exposed to DMF showing (A) mortality, (B) tissue loss, (E) ejection of mesenterial filaments and (F) polyp debilitation. Cumming plots of (C) bleaching and (D) Fv/Fm loss in *M. digitata* exposed to DMF, showing the datapoints with mean and SD, and the mean difference of each concentration to the negative control (NC) with bootstrap 95% CIs. Asterisks indicate statistically significant differences to NC. "PC" indicates the positive control (0.08 mg L^{-1} $CuCl_2$).

Supplement S1.11: Additional Effect Concentration Estimates

Table S11. Overview of acute effect concentrations for all tested substances (Cu^{2+}, BP-3, DCMU, DMF) and endpoints (Mortality, Tissue Loss, Bleaching, Fv/Fm Loss, Filament Ejection, Polyp Debilitation).

Endpoint		Cu^{2+}	BP-3	DCMU	DMF
Mortality	LC_{10}	0.148 (0.121-0.176)	5.0126 (4.299-5.726)	> 9.390	> 1416
	LC_{50}	0.161 (0.055-0.267)	5.716 (5.256-6.176)	> 9.390	> 1416
	NOEC	0.151	5.434	9.39022	> 1416
	LOEC	0.303	6.386	40.93958	> 1416
Tissue Loss	EC_{10}	0.069(0.038-0.099)	4.151 (3.236-5.067)	> 9.390	> 1416
	EC_{50}	0.107 (0.076-0.138)	5.112 (4.604-5.619)	> 9.390	> 1416
	NOEC	0.076	5.434	9.39022	> 1416
	LOEC	0.151	6.386	40.93958	> 1416
Bleaching	EC_{10}	-0.003 (-0.01-0.004)	> 9.078	0.00713 (0.00438-0.00988)	> 1416
	EC_{50}	0.008 (0.005-0.011)	> 9.078	0.02750 (0.01750-0.03751)	> 1416
	NOEC	0.009	> 9.078	0.00441	> 1416
	LOEC	0.019	> 9.078	0.01281	> 1416
Fv/Fm Loss	EC_{10}	> 0.303	> 9.078	0.00793 (0.00598-0.00987)	> 1416
	EC_{50}	> 0.303	> 9.078	0.0242 (0.01972-0.02872)	> 1416
	NOEC	> 0.303	> 9.078	0.01281	> 1416
	LOEC	> 0.303	> 9.078	0.04937	> 1416
Filament Ejection	EC_{10}	> 0.303	> 9.078	> 40.939	8.947 (-2.116-20.011)
	EC_{50}	> 0.303	> 9.078	> 40.939	60.055 (6.460-113.650)
	NOEC	> 0.303	> 9.078	> 40.939	52.44
	LOEC	> 0.303	> 9.078	> 40.939	157.33
Polyp Debilitation	EC_{10}	> 0.303	< 3.087	0.00344 (-0.00136-0.00824)	> 1416
	EC_{50}	> 0.303	< 3.087	0.02582 (-0.00013- 0.05176)	> 1416
	NOEC	> 0.303	0.000	0.01281	> 1416
	LOEC	> 0.303	3.087	0.04937	> 1416

Table S12 Overview of statistical methods used to estimate effect concentration (cf. Table S11). The best fitting Dose Response Model (DRM) was chosen based on the lowest AIC for each individual substance and endpoint.

Endpoint		Cu^{2+}	BP-3	DCMU	DMF
Mortality	LC_{10}/LC_{50}	Weibull (type 1) 2-parameter DRM	Log-normal 2-parameter DRM	-	-
	NOEC/LOEC	Fisher's Exact Tests	Fisher's Exact Tests	Fisher's Exact Tests	Fisher's Exact Tests
Tissue Loss	EC_{10}/EC_{50}	Log-normal 2-parameter DRM	Weibull (type 2) 2-parameter DRM	-	-
	NOEC/LOEC	Fisher's Exact Tests	Fisher's Exact Tests	Fisher's Exact Tests	Fisher's Exact Tests
Bleaching	EC_{10}/EC_{50}	Logistic 3-parameter DRM	-	Weibull (type 1) 4-parameter DRM	-
	NOEC/LOEC	Dunnett's Tests	Permutation Tests	Permutation Tests	Permutation Tests
Fv/Fm Loss	EC_{10}/EC_{50}	-	-	Weibull (type 1) 4-parameter DRM	-
	NOEC/LOEC	Dunnett's Tests	Permutation Tests	Permutation Tests	Permutation Tests
Filament Ejection	EC_{10}/EC_{50}	-	-	-	Weibull (type 1) 2-parameter DRM
	NOEC/LOEC	Fisher's Exact Tests	Fisher's Exact Tests	Fisher's Exact Tests	Fisher's Exact Tests

Polyp Debilitation	EC_{10}/EC_{50}<br><br>NOEC/LOEC	-<br><br>Fisher's Exact Tests	-<br><br>Fisher's Exact Tests	Weibull (type 1)<br>2-parameter DRM<br>Fisher's Exact Tests	-<br><br>Fisher's Exact Tests

References

1. Miller, I.B.; Moeller, M.; Kellermann, M.Y.; Nietzer, S.; Di Mauro, V.; Kamyab, E.; Pawlowski, S.; Petersen-Thiery, M.; Schupp, P.J. Towards the Development of Standardized Bioassays for Corals: Acute Toxicity of the UV Filter Benzophenone-3 to Scleractinian Coral Larvae. *Toxics* **2022**, *10*, 244, doi:10.3390/toxics10050244.
